#  Risk of Bias in Network Meta-Analysis (RoB NMA) tool

**DOI:** 10.1136/bmj-2024-079839

**Published:** 2025-03-18

**Authors:** Carole Lunny, J P T Higgins, Ian R White, Sofia Dias, B Hutton, J M Wright, Areti-Angeliki Veroniki, P F Whiting, A C Tricco

**Affiliations:** 1 Precision, Vancouver, Canada; 2 Knowledge Translation Programme, Li Ka Shing Knowledge Institute, St Michael’s Hospital, Unity Health Toronto, ON, Canada; 3 Cochrane Hypertension Review Group, Therapeutics Initiative, University of British Columbia, Vancouver, BC, Canada; 4 Population Health Sciences, Bristol Medical School, University of Bristol, Bristol, UK; 5 NIHR Applied Research Collaboration West (ARC West) at University Hospitals Bristol and Weston NHS Foundation Trust, Bristol, UK; 6 MRC Clinical Trials Unit at UCL, London, UK; 7 Centre for Reviews and Dissemination, University of York, York, UK; 8 Ottawa Hospital Research Institute, Ottawa, ON, Canada; 9 Ottawa University, School of Epidemiology and Public Health, Ottawa, ON, Canada; 10 Institute for Health Policy, Management, and Evaluation, University of Toronto, Toronto, ON, Canada; 11 Bristol Technology Assessment Group, Population Health Sciences, Bristol Medical School, University of Bristol, Bristol, UK; 12 Dalla Lana School of Public Health and Institute of Health Policy, Management, and Evaluation, University of Toronto, Toronto, ON, Canada; 13 Queen’s Collaboration for Health Care Quality Joanna Briggs Institute Centre of Excellence, Queen’s University, Kingston, ON, Canada

## Abstract

Systematic reviews with network meta-analysis (NMA) have potential biases in their conduct, analysis, and interpretation. If the results or conclusions of an NMA are integrated into policy or practice without any consideration of risks of bias, decisions could unknowingly be based on incorrect results, which could translate to poor patient outcomes. The RoB NMA (Risk of Bias in Network Meta-Analysis) tool answers a clearly defined need for a rigorously developed tool to assess risk of bias in NMAs of healthcare interventions. In this guidance article, we describe and provide a justification for the tool’s 17 items, their mechanism of bias, pertinent examples, and how to assess an NMA based on each response option.

A network meta-analysis (NMA) is a type of quantitative analysis that can be performed as part of a systematic review.^1^ ^2^ ^3^ ^4^ An NMA is an extension of a pairwise meta-analysis that compares the effects of multiple interventions simultaneously on one clinical, public health, or policy question.^1^ NMAs provide coherent estimates of comparative effectiveness for all pairs of interventions in the network, including interventions that have never been previously compared in a head-to-head study. Furthermore, NMAs allow for the ranking of all interventions in a network of studies.^5^

Systematic reviews with NMA have potential biases in their conduct, analysis, and interpretation.^6^ ^7^ Quality assessment of the evidence is integral to the practice of evidence based medicine. If the results or conclusions of an NMA are integrated into policy or practice without any consideration of the risks of bias, decisions could unknowingly be based on incorrect results, with the potential for these to translate to poor patient outcomes. Therefore, NMAs should be assessed in terms of potential for bias.

The Risk of Bias in Network Meta-Analysis (RoB NMA) tool was developed because no tool existed to assess the risk of bias in this type of evidence synthesis. The RoB NMA tool includes three domains containing 17 items, presented as signalling statements, with three overall domain judgments and one summary judgment. To facilitate and promote the use of the RoB NMA tool, we provide a justification for each of the 17 RoB NMA items, their mechanism of bias, pertinent examples, and how to assess the NMA based on each response option. Appendix A provides details about the methods and procedures used to develop the tool, and we provide three examples of RoB NMA assessments in appendix B.

**Summary points**

Researchers, policy makers, healthcare practitioners, and funding bodies need to identify the most trustworthy evidence for decision making
This goal cannot be achieved without a mechanism to evaluate the limitations in the way in which a network meta-analysis (NMA) was planned, analysed, and presented, including the way in which the evidence was assembled
The RoB NMA (Risk of Bias in Network Meta-Analysis) tool is a structured approach for assessing the risk of bias in an individual NMA conducted within a systematic review.
The RoB NMA tool is used after authors publish their NMA, and when external assessors want to determine if the authors conducted the NMA in a way that may bias the NMA findings or conclusions
Descriptions for each of the 17 item statements categorised into three domains is provided, together with a justification for inclusion of the items, source of bias, and illustrative examples
An evaluation of the flaws and limitations of an NMA is crucial in determining whether the study as a whole (including the systematic review portion) is valid
The RoB NMA tool answers a clearly defined need for a rigorously developed tool to assess risk of bias in NMAs of healthcare interventions

## Scope of RoB NMA tool

Many systematic reviews report multiple NMAs, for example in reviews where multiple outcomes are considered. The RoB NMA tool aims to assess the risk of bias in a single NMA within a systematic review. If multiple NMAs are reported for the same outcome, then combined assessment may be possible. As an NMA level tool, RoB NMA is not to be confused with tools to assess the risk of bias in individual primary studies included in systematic reviews, such as the Cochrane risk of bias tool for randomised controlled trials.^8^ Nor should this tool be confused with tools to assess the internal validity of the systematic review within which the NMA is embedded, such as ROBIS (ie, a tool for assessing risk of bias in systematic reviews) for risk of bias^9^ or AMSTAR 2 (A Measurement Tool to Assess Systematic Reviews, version 2) for methodological quality.^10^ The RoB NMA tool should be used with the latter type of tool, as discussed later in this article (section “Use of RoB NMA with other systematic review assessment tools”).

The RoB NMA tool was developed mainly for application to NMAs of healthcare interventions, based on aggregated study level data (and potentially applicable to individual participant data, although testing was not done on NMAs based on individual participant data) (box 1 has definitions and key references). The tool is applicable to both arm based and contrast based data. The RoB NMA tool applies to NMAs comprising randomised controlled trials, non-randomised studies of interventions, or a mixture of both. The tool also covers situations where authors intended to compare multiple treatments in an NMA but then found that the assumptions were violated (eg, a disconnected network, or studies that were too heterogeneous to combine), leading to a decision that an NMA was not feasible.

Box 1 **Definitions and useful references**

**Systematic review**

A systematic review attempts to collate all study specific evidence that fulfils prespecified eligibility criteria to answer a specific research question. It uses explicit, systematic methods that are selected with a view to minimising bias, thus providing more reliable findings from which conclusions can be drawn and decisions made.^24^

**Pairwise meta-analysis**

Pairwise meta-analysis is the type of statistical synthesis, often used in systematic reviews, to combine effect estimates from primary studies comparing one intervention with another.^24^

**Bias in results**

Bias occurs when factors systematically affect the results of a primary study or a systematic review, and cause them to be different from the truth.^24^ The procedures that are required to conduct a meta-analysis or network meta-analysis (NMA) (eg, ensuring that studies are not selectively omitted), or the underlying systematic review (eg, double and independent data extraction), help mitigate the risk of bias in the results. Studies affected by bias can be inaccurate, in particular by overestimating or underestimating the true effect in the target population.

**Bias in conclusions**

A well conducted review draws conclusions that are appropriate to the evidence reviewed, and can therefore be free of bias even when the primary studies included in the review have a high risk of bias. However, bias can be introduced when interpreting the review’s findings. For example, review conclusions may not be supported by the evidence presented, the relevance of the included studies may not have been considered by the authors of the review, and reviewers may inappropriately emphasise results on the basis of their statistical significance.

**Risk of bias**

Risk of bias is the likelihood that aspects of the design, conduct, analysis, interpretation, or reporting of a study will lead to misleading results. An assessment of risk of bias focuses on the potential for study limitations to bias the study findings with respect to the question of interest. Risk of bias is distinguished from the methodological quality of studies (ie, how well the study is conducted) and the reporting quality or comprehensiveness of a published evidence synthesis manuscript (ie, how well authors report their methodology and results). Risk of bias does not mean that the NMA is conclusively biased or that the NMAs themselves are not well conducted.

**Direct comparison**

A comparison of two interventions, for example treatment A versus placebo, or treatment A versus treatment B, is made within studies.

**Direct evidence**

Evidence on the relative effectiveness (or safety) of interventions derived entirely from direct comparisons (ie, based on within study comparisons).

**Indirect comparison**

A comparison of two interventions with one or more common comparators.^3^ ^25^ For example, effect estimates from studies comparing interventions A and C can be combined with effect estimates from studies comparing interventions B and C, to learn about the comparison A versus B.

**Indirect evidence**

Evidence on the relative effectiveness (or safety) of two interventions derived entirely from indirect comparisons. Indirect evidence could be available through various pathways and multiple intermediate comparators (ie, compound indirect evidence).

**Transitivity**

The assumption that an intervention effect measured using an indirect comparison is equivalent to the intervention effect measured using a direct comparison.^4^ In practice, transitivity requires that the sets of studies used to obtain the indirect comparison are sufficiently similar in characteristics that may modify the intervention effects. Transitivity is closely related to statistical consistency, which is the statistical manifestation (in the available evidence) of the transitivity assumption.

**Consistency and inconsistency**

Consistency is the situation in which an intervention effect estimates from indirect evidence does not differ systematically from the intervention effect estimates from direct evidence.^26^ Inconsistency is the converse of this term, and describes a lack of agreement between direct and indirect evidence.^27^ In the presence of mixed evidence, estimating the amount of inconsistency and evaluating it statistically by comparing direct and indirect estimates of the relative effect for the same comparison is possible.^28^ In the context of a random effects meta-analysis, inconsistency usually refers to the mean intervention effect, allowing for the between study variation caused by heterogeneity within the direct evidence.

**Connected and disconnected networks**

**Fig 1:**
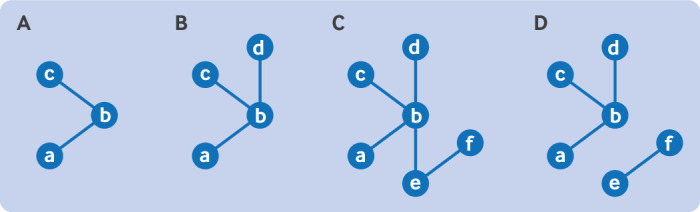
Connected network (A-C) and disconnected network (D). No trials connect intervention E or F to the rest of the interventions, making diagram D disconnected

To conduct an NMA, the interventions should form a connected network, such that a path exists from each intervention to every other intervention in the network.^29^ Figure 1A to C illustrates a connected network, and figure 1D shows a disconnected network, where no trials connect intervention E or F to the rest of the interventions.^30^ ^31^

**Individual participant data NMA**

Individual participant data NMA uses data collected from each participant in each trial in the analysis.^32^ Individual participant data are usually obtained directly from trialists or study sponsors, although the data may be available from study repositories.^33^

**Multi-arm trial**

Multi-arm studies are primary studies with three or more arms comparing different interventions.^34^ ^35^

The intended users of the RoB NMA tool are methodologists who are either epidemiologists or statisticians knowledgeable about bias in NMAs. Guideline developers, health technology assessors, researchers, funders, journal editors, and decision makers (eg, clinicians and policy makers) working alongside methodologists may also find the tool useful. Peer reviewers may find it useful to identify limitations and biases within a systematic review with NMA that is being considered for publication in a journal. The tool could also help other researchers and students of evidence synthesis to judge the validity of an NMA.

## How RoB NMA fits in with other tools

**Fig 2:**
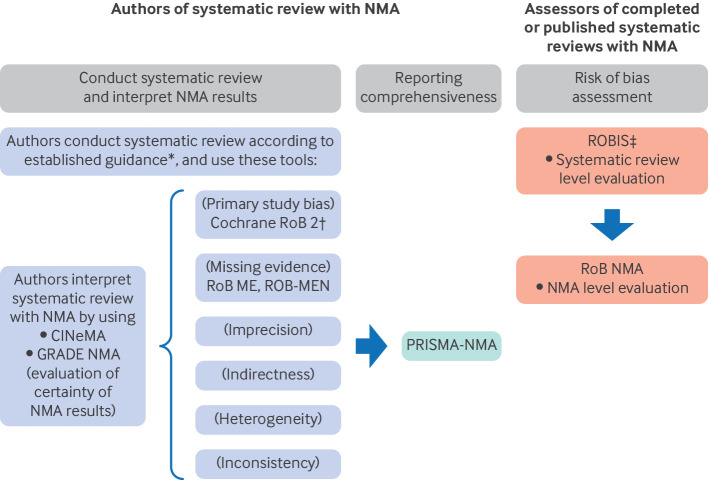
Processes and tools used by authors of systematic reviews with network meta-analysis (NMA), or assessors of completed or published systematic reviews with network meta-analysis. Left: tools that authors use when conducting a systematic review with NMA (eg, Cochrane RoB 2), and when completed and published, assessors can use the tools (right) to assess the systematic review with NMA for biases (eg, ROBIS). Authors of NMAs should first conduct the systematic review using established guidance documents. When all studies have been included, the Cochrane RoB 2 tool can be used to assess the risk of bias of randomised controlled trials (or ROBINS-I for observational studies). RoB-ME and ROB-MEN tools can also be used to evaluate the systematic review with or without NMA. These assessments by NMA authors can then feed into a CINeMA or GRADE NMA assessment of the certainty of the evidence, which includes evaluation of risk of bias, imprecision, indirectness, heterogeneity, and inconsistency. Reporting checklists, such as PRISMA-NMA, help NMA authors when they have completed their review and are ready to publish. The reporting checklists help describe the review and NMA comprehensively, transparently, and accurately. Once the publication or report is complete and publicly available, individuals (ie, assessors) can use the ROBIS and RoB NMA tools to assess the systematic review with NMA for known meta level biases, such as publication bias (eg, where studies are missing from the published literature) and selective non-reporting of outcomes or analyses (eg, because they did not reach a desired level of magnitude or statistical significance). *Established methodological guidance for conducting systematic reviews includes the Cochrane Handbook for Systematic Reviews of Interventions, JBI Manual for Evidence Synthesis, or Cochrane-Campbell Handbook for Qualitative Evidence Synthesis. These guidance documents have detailed steps on how to plan, conduct, organise, and present a systematic review. †Or a suitable alternative for non-randomised studies (ie, ROBINS-I). ‡Or other suitable tools (ie, AMSTAR 2 (A Measurement Tool to Assess Systematic Reviews, version 2)). CINeMA=Confidence in Network Meta-Analysis; GRADE NMA=Grading of Recommendations Assessment, Development, and Evaluation in a Network Meta-Analysis; PRISMA=Preferred Reporting Items for Systematic reviews and Meta-Analyses; ROB ME=Risk of Bias due to Missing Evidence; ROB-MEN=Risk of Bias due to Missing Evidence in Network Meta-Analysis; RoB NMA=Risk of Bias in Network Meta-Analysis; RoB 2=revised Cochrane Risk of Bias tool for randomised trials; ROBINS-I=Risk of Bias in Non-randomised Studies of Interventions

Figure 2 illustrates a range of tools used in evidence syntheses involving NMA. These tools broadly fall into one of two categories: those typically used by authors conducting systematic reviews containing NMAs and those typically used by people using or assessing completed NMAs. Authors of NMAs use a range of tools to guide conduct, interpretation, and reporting. These include tools to assess risk of bias in the included primary studies (RoB 2 (revised Cochrane Risk of Bias tool for randomised trials)^11^), risk of bias caused by missing evidence (ROB ME (Risk of Bias due to Missing Evidence),^12^ and ROB-MEN (Risk of Bias due to Missing Evidence in Network Meta-Analysis)^13^), tools to guide completeness in reporting (PRISMA-NMA (Preferred Reporting Items for Systematic reviews and Meta-Analyses-NMA)^14^), and tools to assess the certainty of the evidence (GRADE NMA (Grading of Recommendations Assessment, Development, and Evaluation in a Network Meta-Analysis)^15^ and CINeMA (Confidence in Network Meta-Analysis)^16^).

The RoB NMA tool is used after authors have published their NMA, and when external assessors want to determine if the authors conducted the NMA in a way that may bias the NMA findings or conclusions. Assessors should use a suitable tool first (eg, ROBIS^9^ or AMSTAR 2^10^) to assess the systematic review portion, and then examine the credibility of the findings from the completed NMA with the RoB NMA tool.

Two out-of-date tools were developed in the early 2010s to evaluate published NMAs. The Professional Society for Health Economics and Outcomes Research (ISPOR) checklist^17^ was developed in 2014 for assessing the relevance, credibility (ie, risk of bias), and reporting comprehensiveness (ie, reporting quality) of a completed NMA. The National Institute for Health and Care Excellence Decision Support Unit (NICE-DSU^18^) checklist for assessing reporting comprehensiveness of completed NMAs was developed in 2011. These tools are more than 10 years old, do not cite the most recent developments in methods, are not comprehensive, and do not focus on risk of bias.

## Overview of RoB NMA tool

**Fig 3:**
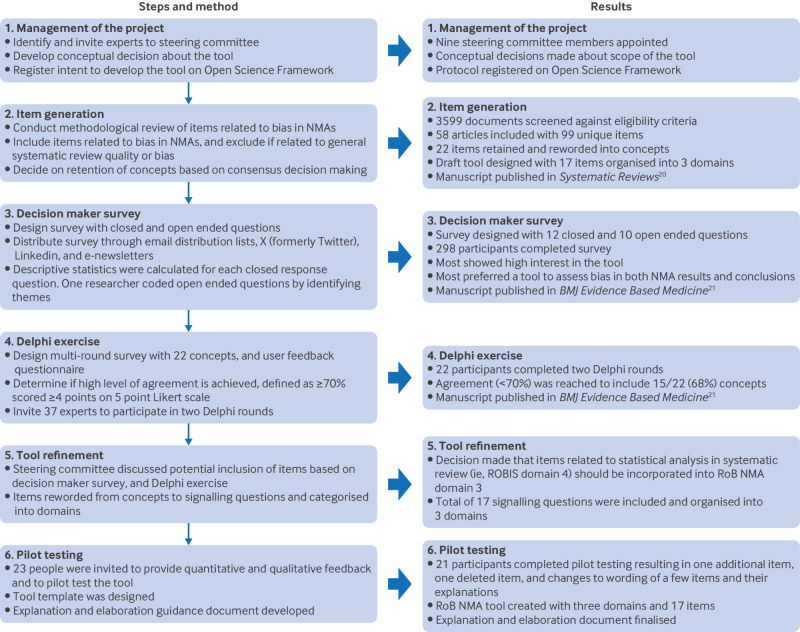
Flow diagram of RoB NMA (Risk of Bias in Network Meta-Analysis) tool development process^20^ ^21^

The RoB NMA tool assesses the potential risk of bias in a single NMA by identifying potential limitations in the way the NMA was conducted, including aspects of how the evidence was assembled that could result in bias in the results or conclusions of the NMA. Whiting et al’s multistep framework for developing quality assessment tools was used as general guidance for tool development.^19^ Our process consisted of steering committee project management, generation of items,^20^ conduct of a decision maker survey,^21^ Delphi exercise,^21^ tool refinement, and pilot testing and refinement (fig 3). Appendix A has a full description of the methods and procedures used to develop the tool.

The tool has 17 items organised into three domains: interventions and network geometry (domain 1), effect modifiers (domain 2), and statistical synthesis (domain 3). NMA results about the ranking of interventions can be affected by various factors, such as unequal number of studies for each comparison in the network, sample size of individual studies, network configuration (eg, few direct comparisons are available), and effect sizes between treatments.^22^ Since these factors are covered in the included items, we have not added a separate item specifically addressing ranking probabilities in the results. However, bias can also be introduced in the conclusions if the authors of an NMA overinterpret rankings, and therefore assessors should consider the potential bias ranking probabilitieswhen interpreting conclusions.

Within each domain is a series of signalling statements. Each signalling statement relates to a feature that may increase the risk of bias in the results or conclusions of the NMA. The response options to the signalling statements are true, probably true, probably false, false, and no information. True responses indicate the lowest risk of bias. When solid evidence exists supporting the signalling statement, the definitive versions (true and false) should be chosen, whereas the probably true and probably false options may be selected when firm evidence is lacking. The no information option should be used sparingly, and only when the assessor believes it is not feasible to make a judgment of true or probably true, or false or probably false. 

Some signalling statements are considered only if preceding statements are judged to be false or probably false. For item 3.9 of the RoB NMA tool, the assessor can also reply, not applicable. Answers to signalling questions and judgments about risk of bias should be supported by written justifications, and quotes from the NMA manuscript.

Judgments on risk of bias for each domain are made based on the evaluation of the signalling statements. Possible judgments at the domain level are low risk of bias, high risk of bias, and some concerns. A judgment of low risk of bias should be assigned only if no concerns are identified across the signalling statements within the domain. A judgment of some concerns indicates that some concerns exist about the potential for bias at the domain level, but not enough to make a definitive judgment. A rating of some concerns can also be assigned to the domain if some potential sources of bias exist that have not been adequately dealt with, and it is not clear whether these sources of bias have influenced the results of the review. Where signalling statements show important potential sources of bias in the NMA, a rating of high risk of bias should be selected. A judgment of no information may differ according to the purpose of the statement item. If the item is expected to be reported (such as exploration of distributions of potential effect modifiers), the absence of information is likely to lead to concerns that a problem exists.

The final phase of the tool combines the RoB NMA judgments with a systematic review level risk of bias or quality judgment (with an appropriate tool, such as ROBIS or AMSTAR 2) to determine whether the NMA as a whole is at risk of bias. Assessors may be interested in whether the results of the systematic review with NMA are at risk of bias, the conclusions of the systematic review with NMA are at risk of bias, or both. The results refer to the collection of quantitative estimates of the relative intervention effects. Bias in these is likely to lead to in bias in other NMA summaries, such as intervention rankings (eg, surface under the cumulative ranking curve (SUCRA)^5^). The conclusions refer to the clinical interpretations drawn from the NMA, which should place the NMA results in the context of all sources of uncertainty about the results. An overall judgment about the individual NMA results follows from domain specific, review level, and NMA level judgments, whereas the overall judgment about NMA conclusions follows from concerns identified at the review level, at the NMA level, and if these considerations are reflected in the author’s interpretations of the conclusions.

During pilot testing, the median time to complete the assessment was 79 minutes (range 30-150 minutes). This time did not include the time to assess the systematic review portion of the NMA (eg, with the ROBIS^23^). 

## Use of RoB NMA with other systematic review assessment tools

**Fig 4:**
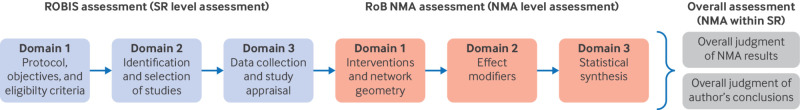
Process for using the ROBIS and then the RoB NMA (Risk of Bias in Network Meta-Analysis) tools to reach overall judgments on two different network meta-analyses (NMAs) in the same systematic review (SR). With the judgments in the first three domains of ROBIS and the three domains of RoB NMA, the assessor then makes an overall judgment about the potential for bias in one or multiple NMA results (eg, one outcome from one network) within an SR. Assessors can also make an overall judgment of the bias in the authors’ conclusions by assessing the interpretation of the findings

The RoB NMA tool is intended to supplement an assessment of the risk of bias in the underlying evidence review, which would ideally be a systematic review. Because systematic reviews with NMA contain many of the same steps as conducting a systematic review with pairwise meta-analysis, assessors should start their assessment of risk of bias with a tool such as ROBIS^9^ ^23^ or another suitable tool (eg, AMSTAR 2^10^). ROBIS is currently the only tool designed specifically to assess the risk of bias in a systematic review (box 2). RoB NMA is designed to work conveniently with ROBIS (box 2 and figure 4).

Box 2 **Use of RoB NMA with ROBIS**

When using the RoB NMA (Risk of Bias in Network Meta-Analysis) and ROBIS tools together, three phases must be completed.

**Phase 1**

Assess relevance/external validity (optional). The extent to which the objectives of the systematic review match the research question being asked by its user (eg, an overview author or guideline developer).

**Phase 2**

Identify concerns with the review process. ROBIS addresses four key domains of study eligibility criteria, identification and selection of studies, data collection and study appraisal, and synthesis and findings. The answers to the signalling questions in the ROBIS domains are yes, probably yes, probably no, no, or no information. RoB NMA encompasses the domain 4 synthesis questions in ROBIS (converted to signalling statements), and therefore this domain is omitted in ROBIS when used with RoB NMA. Having answered the signalling questions in the first three ROBIS domains, assessors can indicate if they have concerns about each domain. Responses are low, high, or unclear concerns.

**Phase 3**

Judge the overall risk of bias in the review. Using the domain based bias judgments in the first three domains of ROBIS and the three domains of RoB NMA, the assessor then makes an overall judgment about the potential for bias in the one network meta-analysis result (eg, one outcome from one network). Assessors can also make an overall judgment about bias in the authors’ conclusions by assessing the interpretation of the findings. This approach examines whether the concerns identified were dealt with, whether the relevance of the studies was considered (ROBIS phase 1), and whether the authors avoided emphasising results based on statistical significance.

## Best practice for using the RoB NMA tool

We recommend that the assessment team has expertise in the conduct of, and biases inherent in, NMAs, as well as clinical expertise in the condition under study to assess signalling statements related to effect modifiers. If an assessor does not have the methodological or clinical expertise to assess the NMA, methodologists or clinicians should be consulted. Any protocol or PROSPERO (International Prospective Register of Systematic Reviews) entry (also called a registered report in areas such as psychology) should be obtained and read together with the systematic review with NMA.^36^

Assessing bias in an NMA when the information is not reported in the manuscript can be challenging. Often the missing information can be retrieved from supplementary files or from the study registration. Attempts can also be made to contact the authors to request additional information or clarification about their methods. A no information rating may be assigned if no information is available about the methods used for a particular item in the tool.

Depending on the aim of the assessment, stopping the assessment may be reasonable if a high risk of bias is found in a particular domain, thus reducing the effort required to reach a judgment about the risk of bias. In some cases, starting with particular statements that are expected to identify high risk of bias may be possible. For example, if a naive approach has been used for the analysis, such as aggregating participants’ outcomes across trials separately for each intervention group (as if the data came from one large, simple trial instead of from multiple trials), then signalling statement 3.1 can be answered as false (ie, randomisation within the study was not preserved). The RoB NMA assessment may therefore be stopped because the NMA results could be judged at high risk of bias. In such a situation, the NMA conclusions are also likely to be at high risk of bias.

## Domain 1: interventions and network geometry

The first domain looks at how the interventions are selected and grouped, and whether they are an appropriate set of interventions for performing an NMA.

### 1.1 All interventions and their comparators included in the NMA are reasonable alternatives for the whole target population

#### Background

To ensure comparability of relative effects across the network, all included participants must be eligible to receive all interventions at the start of the study (including active and placebo, no treatment, control, or usual care, or comparator). This idea is sometimes referred to as joint randomisability.^3^ For example, surgery, chemotherapy, and symptom control treatment are all treatments for non-small cell lung cancer, but these treatments would not be regarded as jointly randomisable because they are suitable for different patient groups: chemotherapy is typically considered after surgery, and symptom control is a strategy for advanced stage disease.

An NMA could be viewed as an attempt to mimic a large randomised controlled trial with one set of eligibility criteria for enrolment, comparing all of the interventions of interest. The closer the participants and interventions in the NMA are to resembling such a hypothetical trial, the lower the risk of bias is likely to be.

#### Source of bias 

If different interventions in the network are suitable for different types of people (eg, some interventions are for first line and others for second line treatment), the likelihood is that participants will differ for different intervention comparisons, leading to failure of the transitivity assumption to hold, and subsequent bias.

#### How to assess this statement

Some interventions have contraindications (ie, the intervention is inappropriate for some types of patients because of their stage of illness, treatment history, adverse effects, or because of expected lower effectiveness). Review authors should state contraindications and show that contraindicated individuals are excluded from all studies. Content expertise is required to judge this information.

For example, a review of convergence insufficiency (an eye condition that affects how the eyes work together when looking at nearby objects) included all non-surgical interventions: home based convergence exercises (pencil or target push‐ups), home based computer vergence or accommodative treatment, office based vergence or accommodative treatment, base‐in prism reading glasses, base‐in prism glasses combined with progressive addition lenses, and placebo or sham intervention.^37^ These interventions are available for all patients with a diagnosis of the condition and patients were therefore considered jointly randomisable.

- Answer true if the review authors provide evidence that all studies include similar participants or that individuals contraindicated for one intervention were excluded from all studies.
- Answer probably true if it seems likely on clinical grounds that studies include similar participants or that individuals contraindicated for one intervention were excluded from all studies, although the authors did not show this informationexplicitly.
- Answer probably false if concerns exist that some participants would not be eligible for some of the interventions.
- Answer false if it is clear that some participants would not be eligible for some of the interventions.
- Answer no information when insufficient data are reported to allow a reasonable judgment to be made.

### 1.2 All eligible interventions were included in the network

#### Background

Empirical research has shown that exclusion of an eligible intervention (either an active intervention or a comparator) from a network can have an important effect on estimates of intervention effects in an NMA.^38^ ^39^ The main concern here is that interventions may be excluded based on their predicted or known effect on the results of the NMA, to ensure that the results agree with a priori views about the relative effects of the interventions.

#### Source of bias 

Exclusion of interventions from the NMA may be because of oversights of the authors, or for pragmatic reasons (eg, resource limitations). The former is unlikely to introduce bias, although it could limit the usefulness of the NMA. The latter may or may not introduce bias, depending on whether the choice of interventions to exclude is related to the results that would have been expected had they been included. Depending on network structure, exclusions could substantially affect the estimated intervention effects, and can affect the ranking of the interventions according to their effectiveness or safety.

#### How to assess this statement

To ensure transparency and completeness of reporting, NMA authors should make it clear what interventions are eligible for inclusion in the NMA and the rationale for this choice. Comparison of the planned (ie, as found in the review protocol) versus the included interventions in the network could reveal inconsistencies, and such inconsistencies should be investigated. NMA authors may provide a list of any studies that have the eligible interventions that were excluded from the NMA, with a rationale for each decision. Examining the rationale for the exclusion of eligible interventions from the network is important to determine whether the decisions are adequately justified. Exclusions may be reasonable (eg, if strong concerns are raised about violating the assumptions of the NMA).

- Answer true if no interventions were inappropriately excluded.
- Answer probably true if interventions were appropriately excluded with clear justification given.
- Answer probably false if interventions were excluded with no clear justification to determine if this action was appropriate or not.
- Answer false if interventions were inappropriately excluded (ie, excluded based on their likely effect on the results of the NMA, to ensure that the results agreed with a priori views about the relative effects of the interventions).
- Answer no information when insufficient data are reported to allow a reasonable judgment to be made.

### 1.3 Interventions were appropriately grouped into nodes in the network

#### Background

Two different studies implementing the same active intervention in an identical way is unusual, just as implementation at two different sites within a study will likely differ. One possible exception might be pharmaceutical interventions examining the same drug in the same formulation at the same dose with the same administration schedule. Grouping of interventions (or specific implementations of interventions) into nodes is therefore often required for an NMA.^40^ This requirement applies similarly to grouping of any control interventions included in the network, including placebos, no treatment, control, or usual care.

Grouping of interventions or their variants is sometimes referred to as lumping (as opposed to splitting) in meta-analysis texts. Three possible approaches can be used by NMA authors to group different interventions or variants in an NMA^41^: (i) a broad lumping approach that groups similar interventions at a broad level and is useful to estimate the effects of intervention classes, (ii) a component lumping-and-dismantling approach informed by meta-regression to investigate effects attributed to different components,^42^ and (iii) a hierarchical (class effect) modelling approach that puts similar interventions together as a class but assumes effect variations between these interventions, using modelling to estimate the effects of specific interventions within the classes.^42^ ^43^ ^44^

#### Source of bias

When interventions are grouped, they should be sufficiently similar (ie, interventions are not expected to differ in their relative intervention effects). Bias could arise for the target question if the effect estimated by the combined interventions does not reflect the effect that would be seen if the target intervention (only, not combined) had been used.

#### How to assess this statement

Evidence of similarity of intervention effects to justify grouping of interventions may come from investigations of heterogeneity in pairwise meta-analyses or even single studies. For example, dose-response analyses may indicate that different doses of an intervention are equivalent, justifying their combination into one network node. The intervention effect estimates may be unbiased provided the dose-response relationship (or time course) is not wrongly specified.

Often insufficient empirical evidence exists to support grouping of interventions explicitly based on similarity of effects. Appropriate clinical expertise may therefore be required to make the judgment. Any quantitative data reported by the authors to support the appropriateness of grouping of interventions should also be considered. Potential grouping of interventions may be beneficial when these decisions are discussed by both clinical and methods experts from the research team, and should ideally be prespecified to reduce the misuse of data analysis to find patterns.

- Answer true if the rationale for grouping interventions was sound, the approach was prespecified, and no suggestion of important differences was evident in the effects of grouped interventions, or if there was no grouping of different interventions.
- Answer probably true if interventions have been combined for which different effects are possible but unlikely.
- Answer probably false if interventions have been combined which are likely to have different effects.
- Answer false if interventions have been combined which are known to have different effects.
- Answer no information when insufficient data are reported to allow a reasonable judgment to be made.

### 1.4 All compared interventions were connected through a suitable chain of within study comparisons

#### Background

Networks of interventions can be disconnected or weakly connected for several reasons.^30^ For example, studies may not have been performed to compare the interventions of interest. Even if comparisons have been made, the studies may not be appropriate to combine into one network. In a disconnected network, some interventions cannot be compared, because a chain of within study comparisons joining them is not present. Efforts to connect an otherwise disconnected network can increase the set of possible intervention comparisons in a network, allowing conclusions to be drawn about the relative effects of a wider range of interventions. Methods must be chosen carefully, however, to avoid the introduction of bias into the synthesis.^31^

#### Source of bias

Some methods that are available to connect an otherwise disconnected network introduce additional evidence at higher risk of bias.^38^ ^45^ ^46^ ^47^ ^48^ For example, non-randomised evidence may be added to a network of randomised controlled trials,^45^ ^46^ or expert opinion may be used to inform one or more comparisons.^38^ ^47^ ^48^

#### How to assess this statement

Authors may have used one of several approaches to connect disconnected or weakly connected networks described in the literature.^30^ ^49^ ^50^ One possibility is to include additional interventions in the network that are not of primary interest.^51^ ^52^ Alternatively, evidence external to the included studies from the main review can be used by authors to bridge the gaps in the network (or strengthen weak links). For example, in a network of randomised controlled trials, observational evidence (eg, registry data) may be brought in for key comparisons that lack evidence from randomised controlled trials,^53^ evidence from other populations may be used,^46^ or expert opinion can be elicited. Authors of NMAs may have used population adjustment methods when poorly connected networks arise because studies examined different populations.^54^

NMA authors may also have dealt with the problem through the analysis model. One option is hierarchical modelling (or class models), which typically assumes that groups of interventions behave similarly,^49^ or component NMA, an approach particularly suited to complex interventions.^55^ The methods and results sections of the NMA should detail whether disconnected networks were encountered during the processes of the review and analyses, and any attempts to connect them. If the authors connected the network by grouping included interventions, this approach should be assessed in item 1.3.

- Answer true if it is clear that the network is connected, or if the network had some disconnected interventions but the authors only made comparisons between interventions in a connected sub-network.
- Answer probably true if the authors connected the network by adding additional interventions for the purpose of creating indirect evidence on the main comparisons of interest.
- Answer probably false if the authors report that steps were taken to connect disconnected interventions with approaches that involved use of observational data, expert opinion, borrowing of data from related conditions, or other such methods.
- Answer false if the network included single arm studies or if the results were reported for disconnected comparisons.
- Answer no information when insufficient data are reported to allow a reasonable judgment to be made.

Assessors can use table 1 to decide what risk to attribute to the review (high/low/some concerns) with the quote from the manuscript, and their rationale for their choice. 

**Table 1:** Summary of concerns regarding domain level network characteristics and geometry (domain 1)

	Judgment and evidence
Judgment response options (choose one of three)	✓ Low risk of bias ✓ Some concerns ✓ High risk of bias
Quotes from the manuscript to support concerns	
Rationale for judgment	

## Domain 2: effect modifiers

The second domain targets effect modifiers. In the context of a meta-analysis, an effect modifier is a study level characteristic that is a cause of heterogeneity in the intervention effects across studies. In an NMA, the transitivity assumption is met when effect modifiers are equivalently distributed across comparisons. Although studies included in a meta-analysis will inevitably differ in many ways, only a subset of these differences is likely to be a result of effect modifiers. Variation in effect modifiers should be kept as low as possible to minimise heterogeneity and ensure the validity of any meta-analysis (pairwise meta-analysis or an NMA).

In an NMA, effect modifiers can also affect the validity of the transitivity assumption, if systematic differences exist in the effect modifiers across the different direct comparisons in the network.^56^ Results of an NMA are generally considered to be more sensitive to differences in effect modifiers between direct comparisons (often called incoherence or inconsistency) than they are to differences within direct comparisons (usually termed heterogeneity). Baker and Kramer provide examples where the transitivity assumption is violated because effect modifiers differ across comparisons.^57^

The first three signalling statements in this domain aim to identify whether important differences exist in effect modifiers across comparisons for three types of effect modifiers separately: outcome definitions and time points, participant characteristics, and study characteristics. Effect modifiers should be sufficiently similar across the studies included, even in a pairwise meta-analysis. Sufficiently similar here means similar in aspects that will influence the effects of the interventions. The fourth signalling statement is used to determine whether the NMA authors could overcome any problems identified by the first three items. The fourth statement is considered only if a problem is identified in one of the first three items.

### 2.1 Outcome definitions and time points were similar across direct comparisons in the network

#### Background

Some degree of variability in outcome definitions and time points is usually to be expected in a meta-analysis. The specific question answered by this signalling statement is whether systematic differences exist in outcome definitions (including measurement methods) or time points (eg, response at six weeks *v* 12 months) across the different direct comparisons made by studies in the network. Differences in outcome definitions or time points can be dealt with in NMA with meta-regression or certain types of mixed effects models. Alternatively, subgroup analyses can be performed to analyse different outcome definitions or time points separately, or sensitivity analysis may show that the effect of including different outcome definitions and time points is minimal.

#### Source of bias

If outcome definitions or time points differ systematically across comparisons in the network in ways that are associated with intervention effects, the assumption of transitivity may not hold. Combining the results with an NMA would then produce incorrect results. For example, if studies comparing intervention A with intervention B mostly measure anxiety and studies of B versus C mostly measure depression, an indirect comparison of these results to estimate the intervention effect A versus C will produce a biased estimate of both the effect on depression and the effect on anxiety if the interventions have different effects on these two outcomes. This finding is because of failure of the transitivity assumption to hold across the sources of direct evidence and is a problem despite the similarity of outcomes within the direct A versus B and A versus C comparisons.

#### How to assess this statement

Inspection of the outcome definitions and time points used in the included studies should be done at the aggregate level of the direct comparisons.^58^ Transitivity can be assessed by comparing the distribution of potential outcome and time point effect modifiers across the available direct comparisons when sufficient data exist. This assessment requires clinical judgment. NMA authors who restrict their primary analysis at one time point are encouraged to perform sensitivity analyses to show how results may be affected by alternative time points.

- Answer true if studies making different comparisons used outcome definitions or time points that were identical, or similar enough for intervention effects not to be affected by these differences.
- Answer probably true if studies making different comparisons used different outcomes or time points and the differences are unlikely to be associated with differences in intervention effect.
- Answer probably false if studies making different intervention comparisons used different outcomes or time points and these are likely to result in different intervention effects.
- Answer false if studies making different intervention comparisons used different outcomes or time points and these are known to result in different intervention effects.
- Answer no information when insufficient data are reported to allow a reasonable judgment to be made.

### 2.2 Effect modifying participant characteristics were similar across direct comparisons in the network

#### Background

The characteristics of participants in included studies should be sufficiently similar across all studies in any meta-analysis. The specific question answered by this signalling statement is whether systematic differences exist in known or suspected effect modifying participant characteristics across the different direct comparisons made by studies in the network. Examples of participant characteristics that could potentially be effect modifiers include age, sex or gender, history of disease, severity of disease, and history of treatment. Valid indirect comparisons can be made, however, if studies differ in participant characteristics that are not effect modifiers. For example, if an intervention works similarly in men and women, including studies with different proportions of men and women in different comparisons may still provide unbiased estimates of the relative effects of the intervention.

#### Source of bias

If effect modifying participant characteristics differ systematically across comparisons in the network, the assumption of transitivity may not hold. Combining the data with an NMA would then produce bias results. For example, if studies comparing intervention A with intervention B mostly include participants with severe illness and studies of B versus C mostly include participants with moderate illness, an indirect comparison of these results to estimate the intervention effect of A versus C will produce a biased estimate of both the effect for severe and moderately ill participants if the interventions have different effects in these two populations. This finding is because of failure of the transitivity assumption to hold across the sources of direct evidence.

#### How to assess this statement

The first step is to identify participant characteristics that are likely to be effect modifying (ie, to affect the extent to which interventions in the network are effective (or safe)). This approach requires clinical judgment and may benefit from reviewing the relevant literature. Potential effect modifiers will ideally be stated a priori in a protocol for the NMA, including method of identification (eg, a systematic review or clinical expert opinion). Inspection of these effect modifying participant characteristics in the included studies should be performed at the aggregate level of the direct comparisons to ensure that the assumption of transitivity is plausible.^58^ This approach may also require clinical judgment. Information about effect modifiers may be available in tables detailing the included studies or from a thorough descriptive comparison of differences in patient characteristics. Good examples are found in a 2024 empirical study by Spineli and colleagues.^59^

- Answer true if studies making different comparisons were similar in known and suspected effect modifying participant characteristics.
- Answer probably true if studies making different comparisons were reasonably similar (or are judged to be reasonably similar) in known or suspected effect modifying participant characteristics.
- Answer probably false if suspicions arise that effect modifying participant characteristics were not reasonably similar across comparisons.
- Answer false if evidence exists of important variation across comparisons in known or suspected effect modifying participant characteristics.
- Answer no information when insufficient data are reported to allow a reasonable judgment to be made.

### 2.3 Effect modifying study characteristics were similar across direct comparisons in the network

#### Background

Examples of study characteristics that could potentially be effect modifiers include study design features (such as whether cluster randomisation or a run-in period was used), study setting (eg, primary *v* secondary care), or study conditions (eg, summer *v* winter in respiratory infections). The specific question answered by this signalling statement is whether systematic differences exist in effect modifying study characteristics across the different direct comparisons made by studies in the network.

If NMA assumptions (ie, transitivity or consistency) do not hold, the findings could be biased. The assumptions underlying the statistical analysis provide the framework for the validity of the results obtained. Violation of these assumptions could affect the accuracy or produce incorrect results. Valid indirect comparisons can be made, however, if studies differ in study characteristics that are not effect modifiers. For example, the use of clustering may not affect intervention effects, and in this case it does not matter if A versus B studies mostly used clustering and A versus C studies mostly did not.

#### Source of bias

Effect modifying study characteristics can vary across studies for a particular comparison (as with standard pairwise meta-analysis, causing heterogeneity), but also between comparisons (causing inconsistency) in the network.^56^ ^60^ If an imbalance in the distribution of effect modifiers between different types of direct comparisons exists, the NMA will be biased. For example, if studies comparing intervention A with intervention B are mostly conducted in primary care and studies of B versus C are mostly conducted in secondary care, an indirect comparison of these results to estimate the intervention effect A versus C will likely be biased for either of these settings, if intervention effects tend to differ between settings. This finding is because of failure of transitivity (or consistency) to hold across the sources of direct evidence.

#### How to assess this statement

The first step is to identify study characteristics that are likely to be effect modifying. This approach is likely to require clinical judgment. The most likely of these are specific design features that are associated with different magnitudes of effect. Potential study level effect modifiers will ideally be stated a priori in a protocol for the NMA (eg, separate systematic review or expert input).

Inspection of these effect modifying study characteristics in the included studies should be done at the aggregate level of the direct comparisons to ensure that assumptions of transitivity are plausible. Information about effect modifiers may be available in tables detailing the included studies or from a thorough descriptive comparison of differences in studies making different direct comparisons. This item does not include the risk of bias at the primary study level, which is dealt with in statement 3.3.

- Answer true if studies making different direct comparisons were similar in known and suspected effect modifying study characteristics (including consideration of the nature and direction of any biases).
- Answer probably true if studies making different direct comparisons were reasonably similar (or are judged to be reasonably similar) in most known or suspected effect modifying study characteristics.
- Answer probably false if suspicion arises that effect modifying study characteristics were not reasonably similar across direct comparisons.
- Answer false if evidence exists of important variation across direct comparisons in known or suspected effect modifying study characteristics.
- Answer no information when insufficient data are reported to allow a reasonable judgment to be made.

### 2.4 If false or probably false to 2.1, 2.2, or 2.3: the analysis appropriately looked at the differences in effect modifiers across the network

#### Background

Differences in effect modifiers across different parts of the network can lead to a lack of transitivity in the network, which may not be identified by standard tests for inconsistency.^60^ Conversely, systematic differences in effect modifying characteristics across the different direct comparisons made by studies in the network do not result in bias if they are accounted for in the analysis. When a network does not have many closed loops, global statistical tests for inconsistency may not be informative, and loop based inconsistency checks should be preferred, although they generally have low statistical power to detect inconsistency.^26^ When formal statistical tests are inconclusive or cannot be performed (eg, in the absence of closed loops in the network), evaluation of the transitivity assumption relies on the non-statistical consideration of the similarity of effect modifying characteristics, and this information should be described in sufficient detail.

#### How to assess this statement

Meta-analysts may have tackled the problem with appropriate statistical methods (eg, meta-regression, subgroup analysis, or mixed effects models) that were likely to account for these differences, or sensitivity analysis showed that the differences were unimportant. Potential effect modifiers may have been measured at the individual participant level or the study level. Potential effect modifying participant level characteristics will ideally have been measured at the individual level. An appropriate network meta-regression then requires individual participant data. Participant level characteristics may have been aggregated at the study level, but this finding introduces a risk of aggregation bias.

- Answer true if the authors have used acceptable methods to conduct the analysis, such as statistical methods (eg, meta-regression, subgroup analysis, or mixed effects models) that were likely to account for these differences, or sensitivity analysis showed that the differences were unimportant in the review, adjusting for the inclusive set of effect modifiers in question, and dealt with the comparability of findings with those from unadjusted analyses.
- Answer probably true if analyses were partly explored, but differences remain that were not dealt with or if findings from analyses were not adequately considered.
- Answer false or probably false if analyses to look at differences in potential effect modifiers were not explored.
- Answer no information when insufficient data are reported to allow a reasonable judgment to be made.

Assessors can use table 2 to decide what risk to attribute to the review (high/low/some concerns) with the quote from the manuscript, and their rationale for their choice. 

**Table 2:** Summary of concerns regarding domain level network characteristics and geometry (domain 2)

	Judgment and evidence
Judgment response options (choose one of three)	✓Low risk of bias ✓Some concerns ✓High risk of bias
Quotes from the manuscript to support concerns	
Rationale for judgment	

## Domain 3: statistical synthesis

The last domain looks at the statistical synthesis, covering aspects such as non-reporting biases, biases within studies, statistical methods, inconsistency (conflict) between direct and indirect evidence, and sensitivity analyses. Signalling statement 3.8 is considered only if signalling statement 3.7 is judged to be false or probably false. In this domain, NMA authors can use sensitivity analysis to deal with shortcomings in the main analysis that would result in a probably true, probably false, or false rating. If a sensitivity analysis was performed which would have received a higher rating, that higher rating can be assigned if the sensitivity analysis gave results similar to the main results.

### 3.1 The analysis respected within study randomisation

#### Background

A simple but inappropriate statistical method is to compare the results of individual arms from different studies as if they were from one study. A naive analysis that ignores within study randomisation implicitly uses comparisons between studies as well as within studies.^61^ ^62^ An example of an unadjusted indirect comparison approach, which combines trial data as if the data came from one large trial, is a comparative review of the tolerability of roxithromycin and erythromycin in patients with lower respiratory tract infections.^63^ The authors ignored within study randomisation, and used an unadjusted indirect comparison approach, combining trial data as if the data came from one large trial. In this case, the authors erroneously reported adverse events based on data from arms of all randomised controlled trials (both head-to-head and indirect comparisons).

Analysis of randomised trials should always be based on comparisons of comparable groups. Therefore, an NMA of randomised trials should also draw conclusions based on the randomised evidence (ie, on the comparisons within studies). An NMA of observational studies should similarly be based on within study comparisons because these are likely to have attempted to control for confounding factors.

#### Source of bias

Comparisons between studies may be biased by even small differences in overall outcome between studies, arising for example from small differences in patient groups or outcome definitions.

#### How to assess this statement

Combining arms across studies (for each intervention) and comparing the overall arm results across interventions in the network does not respect the randomisation and would be an inappropriate approach. Older studies may be more prone to this approach because the methods for NMAs were not fully developed and popularised. Any analysis that uses comparisons within study as data respects the randomisation. Analysis of arm level or patient level data may or may not preserve the randomisation. The analysisrespects the randomisation if a fixed study effect is included in the statistical model. Arm based methods of analysis include a random study effect in the statistical model^64^ and may make some use of comparisons between studies.^65^ This approach is likely to be a source of bias if average outcomes were different across direct comparisons in the network.^65^

- Answer true if within study comparisons were analysed, or arm level or patient level data were analysed with a model including a fixed effect of study.
- Answer probably true if arm level or patient level data were analysed with a model including a random effect of study, and average outcomes were similar across direct comparisons. Also answer probably true if this approach was used in a sensitivity analysis which gave results similar to the main results.
- Answer probably false if arm level or patient level data were analysed with a model including a random effect of study, and average outcomes were different across direct comparisons.
- Answer false if arm level or patient level data were analysed ignoring a model that includes a random or fixed study effect, or if data were combined across studies before being compared between interventions.
- Answer no information when insufficient data are reported to allow a reasonable judgment to be made.

### 3.2 No publication bias or other selective non-reporting biases were suspected

#### Background

Like any meta-analysis, an NMA should include all studies that have collected data relevant to the question being raised. Missing whole studies, specific results (eg, outcomes), and unfavourable analyses from an NMA can be influenced by the P value, or direction or magnitude of the study’s effects. A typical example is that significant results suggesting that a treatment is effective are more likely to be published than non-significant results. Studies with significant results are also more likely to be rapidly available, published in high impact journals, and cited by others, and hence more easily identifiable for systematic reviews. Bias in selection of the reported result, where the authors of the study select a result for reporting from among multiple measurements or analyses, on the basis of the P value, or magnitude or direction of the results, is different than bias due to missing results. The term reporting bias has often been used to describe this problem, but we will use the term non-reporting bias here.

This item looks at situations in which a study, result, or analysis may not be reported for several reasons:

- a study was performed but not published;
- the relevant result from an included study is not available to the review authors;
- the review authors have unintentionally failed to collect or process the data available; or
- the review authors have intentionally excluded the result or an analysis from the NMA.

#### Source of bias

Missing primary studies or results may cause bias in the estimates of the effect of the intervention.^66^ ^67^ The important consideration here is whether the study result was not reported because of the finding (eg, magnitude, direction, or P value of the result), in which case omission of the study result will introduce bias into the NMA. This bias is often referred to as publication bias when studies are not published, or (within studies) selective reporting bias when undesirable results or analyses are concealed and not published. If a result is excluded for reasons unrelated to the finding (eg, solely for practical reasons, such as paper length), the NMA may lose precision but should not be at risk of bias. Review authors may make inappropriate decisions to exclude some results from the NMA. One potential example would be exclusion of studies considered to be responsible for a large degree of between study heterogeneity based on statistical considerations alone (although these exclusions may be reasonable in a sensitivity analysis).

#### How to assess this statement

Determining whether some studies were not identified by the systematic review is challenging. Comparison adjusted funnel plots^68^ and related statistical analyses can provide clues as to the possibility of reporting bias within and across the studies. The assessor should also examine the numbers of studies identified for potential inclusion in the NMA (eg, from a flowchart within an underlying systematic review) and the numbers of studies actually included in the NMA, together with any reasons provided for excluding studies or results. A mismatch in numbers would result in consideration of why relevant results are not included in the NMA.

- Answer true if all studies were likely to have been identified and all relevant results from identified studies were included in the NMA or it is clear that any results not included in the NMA were excluded for reasons unrelated to the findings (eg, because the studies did not measure the outcome of interest).
- Answer probably true if the amount of evidence potentially excluded from the NMA is so small that its inclusion would have a trivial effect on the findings, or if any results not included in the NMA were likely excluded for reasons unrelated to the findings.
- Answer probably false if eligible results missing from the NMA is likely (because of suppression by the study authors or exclusion by the review authors) and either may be systematically different from the results included in the NMA or the amount of evidence missing from the NMA is sufficiently large that its inclusion could affect estimated intervention effects from the NMA.
- Answer false if evidence exists that eligible results were missing from the NMA (because of suppression by the study authors or exclusion by the review authors) and these results were likely to be systematically different from the results included in the NMA, with a sufficient amount of evidence missing that it could affect estimated intervention effects from the NMA.
- Answer no information when insufficient data are reported to allow a reasonable judgment to be made.

### 3.3 All predefined analyses, and only those analyses, were reported, or discrepancies were explained

#### Background

The NMA should have followed a published or accessible protocol or statistical analysis plan. Examples are protocols registered with PROSPERO (www.crd.york.ac.uk/PROSPERO/), protocols published online, or protocols deposited in an online repository. A protocol could prevent subjective decision making about how the NMA is conducted and what data are included. Without a protocol, decisions made about what studies are included, what data are extracted, and how the data are analysed are likely to be made in light of the data.^69^ With the various results reported for each trial included in a meta-analysis, authors could purposefully select a favourable or unfavourable result from each study. By selecting the best results in this way, reviewers who pick studies to be included could bias findings towards a preconceived assumption.^70^ For instance, in a methods study examining results for the effects of the drug gabapentin for pain, the authors could manipulate the findings such that the meta-analyses with the most extreme effects in either direction made the drug seem either highly effective or completely ineffective for treating neuropathic pain.^70^ ^71^ Although methods described in a protocol should generally be followed, deviating from the planned methods is sometimes necessary. Any such changes should be done in ways that do not introduce bias and should be fully explained.

#### Source of bias

Biases may be introduced by the review authors through their selection of analyses, analysis methods, and data such that unfavourable results are concealed or replaced. 

#### How to assess this statement 

The protocol (ie, preplanned or registered report) should be obtained and read together with the NMA to determine if analyses have been omitted.

- Answer true if predefined analyses were clearly followed (eg, because a detailed, prespecified protocol was followed).
- Answer probably true if there is an indication that predefined analyses, and only those analyses, were followed (eg, because a protocol is insufficiently detailed but the methods implemented are sensible and in accordance with what was written in the protocol), no protocol is available but the methods section seems rigorous and all analyses mentioned are dealt with in the results, or all deviations from the planned methods were justified with reasons unrelated to the observed results.
- Answer probably false if there is no indication that predefined analyses were followed, for example because of insufficient detail about the methods that are likely to have been planned and implemented (eg, no protocol and insufficient clarity in the methods section).
- Answer false if predefined analyses were clearly not followed, or that other analyses were used, and an indication (or evidence) that the deviations from the predefined analyses were made because of the results.
- Answer no information when insufficient data are reported to allow a reasonable judgment to be made.

### 3.4 Biases in primary studies were minimal or addressed in the synthesis

#### Background

NMAs including studies at high risk of bias are themselves at risk of bias, and may lead to an overestimation or underestimation of intervention estimates of effect.^72^ Therefore, assessing the potential for biases in the results of the included primary studies is important. In an NMA, understanding how studies informing particular contrasts affect bias in intervention effects for other contrasts is important. Understanding how studies informing particular contrasts depends on the structure of the network and may be complex to evaluate fully.^73^

#### Source of bias

Flaws in the conduct of randomised trials 74 and other primary study types can result in biased estimation of the intervention effect in a network of interventions.

#### How to assess this statement 

Review authors should have assessed the risk of bias with an appropriate tool, such as the Cochrane RoB 2.0 tool for randomised trials.^11^ Valid conclusions can sometimes be drawn from NMAs that include results judged to be not at low risk of bias, which is partly because a study at high risk of bias may not give biased results, and partly because it can sometimes be established that particular studies have no effect on the conclusions. Sensitivity analyses or threshold analyses^75^ may be used to show, for example, that exclusion of studies at higher risk of bias does not change the estimated intervention effects.^73^ Alternatively, methods to adjust for bias in NMAs are available. The main approaches are those that incorporate direct adjustments for bias into individual studies (eg with prior distributions for biases)^76^ ^77^ or are specific to the context of an NMA and can estimate and adjust for bias with only the included data.^78^ If the NMA authors assessed the certainty in their body of evidence (eg, with GRADE-NMA^79^ or CINeMA),^16^ this assessment does not justify a true or probably true answer here.

- Answer true if all studies were assessed as being at low risk of bias or sensitivity analyses showed that including studies at higher risk of bias had no effect on the results.
- Answer probably true if the proportion of information at high risk of bias was too small for it to affect the results, sensitivity or threshold analyses showed that including studies at high risk of bias had a minimal effect, or adjustment approaches were used that are likely to have corrected for biases.
- Answer probably false if the proportion of information at high risk of bias was sufficient for it to affect the results and sensitivity analyses did not show that including studies at high risk of bias had a minimal effect, bias adjustment approaches were used and whether they are likely to have corrected for biases is unclear, or risk of bias in the included studies was not assessed.
- Answer false if important biases exist in primary studies and these have not been dealt with by the reviewers or sensitivity analyses show that results are strongly influenced by studies at higher risk of bias.
- Answer no information when insufficient data are reported to allow a reasonable judgment to be made.

### 3.5 Appropriate methods were used to handle multi-arm studies

#### Background

If an NMA includes studies that (individually) compare three or more interventions (multi-arm studies), ensuring that these are dealt with appropriately is important.^34^ ^35^ Two aspects can introduce bias: how the multi-arm studies are represented in the data and how they are analysed in the statistical model. The key concern in the latter is how random effects are modelled.

In handling study level data, NMA can incorporate evidence from multi-arm studies by retaining the original multi-arm structure of the study and adequately accounting for the correlation in effects across arms.^80^ When arm level data are used in the analysis (typically only done in the bayesian framework), including multiple arms separately is unlikely to be a source of bias. An alternative is to generate pairwise comparisons within the multi-arm studies before doing the NMA, and to include these pairwise comparisons (contrasts) in the dataset. Including multiple contrasts from one study in a contrast based model will not be a source of bias if the correlations between those contrasts are explicitly accounted for.^81^ ^82^ If these correlations are ignored, however, interventions that occur in more than one contrast will be given unwarranted influence on the NMA. When a random effects model is used, the method of analysis should adequately account for the correlation between the multiple random effects that are estimated from a multi-arm study.

#### Source of bias

The arms in a multi-arm study are correlated and not independent. If NMA authors ignored the dependence of the arms, the variances of the estimates of effect would be underestimated, and biases in effect estimates potentially exaggerated because of incorrect weights.^82^ Ignoring the correlation would also likely underestimate the inconsistency of the whole network.^81^ Incorrectly accounting for correlations, however, could result in incorrectly calculating confidence or credible intervals, which may result in bias in the conclusions.

#### How to assess this statement

Both the handling of the study level data and modelling of random effects should be assessed. For handling of study level data, information about how multi-arm primary study data were extracted and included in the network should be available in the data analysis section of the NMA report. If a multi-arm study is converted into a two arm study by collapsing active arms into one arm, this action is unlikely to be a source of bias, provided that the collapsed arm corresponds to one intervention node in the network (see item 1.3). If a control intervention arm is divided to form several two arm studies, this action is likely to introduce bias in the standard errors of results of the NMA,^24^ because incorrect calculation of the standard errors could have implications for the weights and hence the point estimate or confidence intervals obtained. When data are in contrast level format, the assessor should look to see if the contrasts were formed without dividing the control arms and the meta-analysts correctly specified the correlations in the multiple contrasts from multi-arm trials used in the synthesis model. This finding could be especially the case if multi-arm trials contribute most of the evidence in a network or on particular comparisons, as may be the case if many of the studies are exploring different doses.

In modelling of random effects, it is important that the chosen analysis method accounts for the correlation in random effects from multi-arm studies. Accounting for the correlation applies to both arm based and contrast based models where random effects models are used. Statistical software or code may incorporate these methods for handling multi-arm studies, such as the WINBUGS^83^ ^84^ code reported by Dias et al.^80^ Several R packages provide functionality for NMAs, including the handling of multi-arm trials. Popular packages include gemtc, netmeta, netmetaXL, and rjags (for use with JAGS(Just Another Gibbs Sampler)). This list is not exhaustive and those unfamiliar with similar packages should consult a statistician.

- Answer true if no multi-arm studies exist, as clearly indicated by descriptions of the included studies. Also answer true if the data from multi-arm studies were included either as arm level data or as relative effects with an adequate correlation supplied and the methods used adequately account for the correlation in random effects from multi-arm studies (if a random effects model was used).
- Answer probably true if NMA software, code, or methods were not fully reported to deal with the correlation in random effects in multi-arm studies, but other evidence suggests appropriate software and methods were used.
- Answer false or probably false if multi-arm studies were not appropriately represented in the data set or the methods chosen did not adequately account for the correlations in random effects or did not indicate the appropriate handling of these correlations.
- Answer no information when insufficient data are reported to allow a reasonable judgment to be made.

### 3.6 Appropriate assumptions were made about homogeneity or heterogeneity of effects within comparisons

#### Background

Many different assumptions can be made in an NMA about heterogeneity of intervention effects. By heterogeneity we here refer to variation in intervention effects within a specific intervention comparison. Four approaches can be considered:

- assume that the underlying intervention effect is the same (homogenous) for all studies of a particular comparison, and that this underlying effect applies to every comparison (common effect or fixed effect NMA model);
- assume heterogeneity of effect exists within comparisons, and force the amount of heterogeneity to be the same for every comparison (restricted random effects model);
- assume that heterogeneity of effect within comparisons may exist, and allow this heterogeneity of effectto be different for different comparisons (flexible random effects model); and
- assume that the underlying intervention effect is the same for all studies of some comparisons, but allow heterogeneity of effect across studies of other comparisons (hybrid model).

The most common approaches are the fixed effect model and the restricted random effects model. The latter requires estimation of only one quantity to represent heterogeneity. If the assumption made about heterogeneity is wrong, the results of the NMA can be affected. If heterogeneity is wrongly assumed to be absent, it will not be reflected in inferences, which may seem to be more precise than they should be. If heterogeneity is allowed for but is wrongly assumed to be the same for all comparisons, inferences for comparisons with larger (true) heterogeneity may seem more precise than they would be under a flexible random effects model, whereas inferences for comparisons with smaller (true) heterogeneity may seem less precise than they would be. With few studies for each comparison, heterogeneity may be poorly estimated, which in turn could inflate the uncertainty.

Mismatches between assumptions and data affect the weights given to studies in the NMA and therefore some sources of indirect evidence may be given inappropriately large or small weights. If unexplained heterogeneity is present, meta-regression may be used to investigate whether it can be explained by study level or intervention level characteristics. For example, heterogeneity across studies implementing a drug at different doses may be explained by including dose as a covariate in a network meta-regression. If heterogeneity is adequately explained by meta-regression, the choice between different fixed effect and random effects models will be less important.

#### Source of bias

Heterogeneity in an NMA can result in different estimates of intervention effects when different homogeneity or heterogeneity assumptions are chosen. Models that give different weighting schemes to large and small studies in the NMA can exaggerate biases (eg, those in smaller studies). Inappropriate homogeneity or heterogeneity assumptions therefore imply a risk of bias because studies may have been chosen by the NMA authors based on the results. Inappropriate homogeneity or heterogeneity assumptions often also result in incorrectly estimating confidence or credible intervals. Inappropriate homogeneity or heterogeneity assumptions should be taken into account when assessing bias in the conclusions of the NMA.

#### How to assess this statement

Information on assumptions made about heterogeneity in the analysis model should be available in the data analysis section of the NMA report. Looking at the nature of the heterogeneity among the studies identified is also useful, which may be available from forest plots of direct comparisons. Often some direct comparisons will be presented with large numbers of studies (which are informative about heterogeneity) and other comparisons with few studies (which are usually not informative about heterogeneity). Emphasis should be placed on comparisons with larger numbers of studies.

- Answer true or probably true if heterogeneity is not modelled (eg, with a fixed effect model for rare events) and this approach is well justified, or if unexplained heterogeneity is modelled (ie, random effects model) and no evidence exists in the data against the modelling assumptions. One justification for a particular modelling approach is if a sensitivity analysis is performed with an equally appropriate alternative or more general modelling approach and this sensitivity analysis shows results similar to the main results.
- Answer false or probably false if heterogeneity is not modelled and this approach is not well justified, or if heterogeneity is modelled and evidence exists in the data against the modelling assumptions.
- Answer no information when insufficient data are reported to allow a reasonable judgment to be made.

### 3.7 No evidence of conflict between direct and indirect estimates of the same effect

#### Background

An NMA is not valid when the direct and indirect evidence are not in agreement. Conflict between direct and indirect evidence implies that the fundamental assumption of transitivity in the NMA does not hold. Exploring the evidence for such conflict is an important part of an NMA. Signalling statements 2.1-2.3 looked at whether the nature of the studies makes transitivity plausible. This signalling statement answers whether the intervention effects offer any evidence about transitivity.

Consistency (the statistical manifestation of transitivity) can be assessed only when closed loops are in the network. A closed loop is defined as a set of contrasts for which independent sources of direct and indirect evidence are available.^85^ Loops formed only of comparisons from a multi-arm study are inherently consistent and do not need to be examined, because transitivity must hold within one multi-arm study.

Evidence about conflicts between direct and indirect sources of evidence may come from local investigations of inconsistency (eg, node splitting) or global investigations of inconsistency (eg, comparisons of fit of consistency and inconsistency models to the data, design-by-treatment test). A problematic way to identify conflict is to compare indirect estimates with NMA estimates, because the indirect evidence is included in both estimates. Comparing indirect estimates with direct estimates is better because the estimates are statistically independent. In some networks, suspicions may be raised that a particular closed loop or a particular region of the network has a higher likelihood of inconsistency. In this case, the assessment of conflict should include one or more assessments local to that loop or region. Otherwise, inconsistency can be assessed across the whole network with either multiple local methods or a global method.

#### Source of bias

When direct and indirect evidence for a comparison do not agree, that comparison has inconsistent information. Depending on which source of evidence is nearer the truth for this comparison for the underlying research question raised by the network, and on the relative amount of information in each source, results for this comparison and other comparisons informed by it may be biased.^3^ ^26^

#### How to assess this statement

In determining whether evidence of inconsistency exists, considering the methods used to identify inconsistency is important. Assessors should examine whether the meta-analysts appropriately analysed any closed loops, and whether the results were interpreted by taking into account the network structure, direction of effects, and uncertainty in the estimates. The global method has been found to be underpowered to detect inconsistency.

- Answer true if no potential sources of inconsistency in the network exist (ie, no closed loops or loops formed only by the comparisons in a multi-arm study), or if a suitable exploration of inconsistency was applied with no indication that inconsistency was present (this approach would usually require a sizeable evidence base to overcome problems of low power in tests for inconsistency).
- Answer probably true if a suitable exploration of inconsistency was applied and no evidence of inconsistency exists. This approach could include cases where some estimates are extreme because of zero cells, for example, but the overall direction of effect is consistent.
- Answer probably false if some evidence of inconsistency exists but was not serious, or important concerns were raised that the tests performed had low power to detect inconsistency.
- Answer false if no information about inconsistency is provided, or clear evidence of inconsistency exists.
- Answer no information when insufficient data are reported to allow a reasonable judgment to be made.

### 3.8 If false or probably false to 3.7: conflicting results between direct and indirect evidence were adequately dealt with

#### Background

Conflict between direct and indirect evidence may lead to bias in network results. Suitable ways to avoid bias can include careful re-examination of study and population characteristics (including risk of bias and node definition) to identify differences in studies comparing different interventions. Alternative analyses, such as subgroup analysis or sensitivity analysis, lumping or splitting nodes, or considering alternative assumptions (eg, fitting a random effects model instead of a fixed effect model or adjusting for covariates through meta-regression) can be considered. However, these analyses should be considered exploratory analyses. Also, these steps must have an adequate justification and additional analyses should have been outlined in the protocol.

#### How to assess this statement

The assessor may look at how the meta-analysts examined conflicts between direct and indirect evidence, and judge whether these were appropriately explored. A particularly problematic approach is to omit individual primary studies until the inconsistency is removed (ie, until the P value for inconsistency drops below a threshold for statistical significance). Methods to deal with inconsistency include using a model that explicitly allows for inconsistency (such as the design-by-treatment interaction model and network meta-regression model). The appropriate approach will depend on the nature and extent of the inconsistency, the available data, and the research question being raised.

- Answer true or probably true if the alternative analyses had a clear justification, and resolved the original conflict.
- Answer false or probably false if the direct meta-analysis results were compared with NMA results or previous literature (eg, systematic reviews) to deal with conflicting results, or if the primary studies were randomly excluded until consistency was reached.
- Answer no information when insufficient data are reported to allow a reasonable judgment to be made.

### 3.9 If a bayesian analysis was performed, the choice of prior distributions was appropriate

#### Background

Bayesian analysis requires prior distributions for unknown parameters, including the heterogeneity variance or variances, and the intervention effects. These prior distributions can be informative or vague (non-informative). Informative prior distributions can be based on previous knowledge (eg, with empirical distributions to specify a prior distribution for the heterogeneity variance^86^ ^87^ ^88^ or with observational data external to the NMA^89^ to specify a prior distribution for the intervention effects). The choice of prior distributions should be justified and appropriate, particularly when only a few studies for each comparison or few connections (comparisons) and loops in the network exist. If an informative prior distribution is used, the source of the information should be stated and justified. An informative prior distribution on the intervention effects can only be justified in exceptional circumstances.

#### Source of bias

Inappropriate prior distributions can cause bias, particularly when evidence is sparse, or if they represent incorrect assumptions (eg, are too restrictive) or incorporate evidence or beliefs that are untrue or unrealistic. Some inappropriate prior distributions (eg, for heterogeneity variance) may lead to in incorrect confidence or credible intervals, although not point estimates, which may cause bias in the conclusions.

#### How to assess this statement 

When prior distributions are claimed to be non-informative, the assessor may want to assess the range of prior distributions in relation to prior expectations for the parameters and the amount of information in the network, as reported by the meta-analysts. The assessor can look at the posterior densities, if reported, to ensure that no artificial truncation is present and that they are sufficiently informed by the data. When prior distributions are informative, the assessor may consider whether they are grounded in data that are relevant to the NMA being considered. For example, informative prior distributions for heterogeneity parameters should be based on evidence from similar types of studies in terms of intervention comparisons and outcomes.^90^

- Answer not applicable if a bayesian analysis was not conducted (ie, frequentist analysis).
- Answer true if prior distributions were used that were clearly non-informative across the range of possible parameter values, if informative prior distributions were used and had a clear justification, or if a sensitivity analysis with alternative appropriate prior distributions was performed and showed results similar to the main results.
- Answer probably true if off-the-shelf non-informative prior distributions were used.
- Answer false or probably false if informative prior distributions were used that were not clearly justified.
- Answer no information when insufficient data are reported to allow a reasonable judgment to be made.

Assessors can use table 3 to decide what risk to attribute to the review (high/low/some concerns) with the quote from the manuscript, and their rationale for their choice. 

**Table 3:** Summary of concerns regarding domain level network characteristics and geometry (domain 3)

	Judgment and evidence
Judgment response options (choose one of three)	✓Low risk of bias ✓Some concerns ✓High risk of bias
Quotes from the manuscript to support concerns	
Rationale for judgment	

## Overall risk of bias in NMA

**Fig 5:**
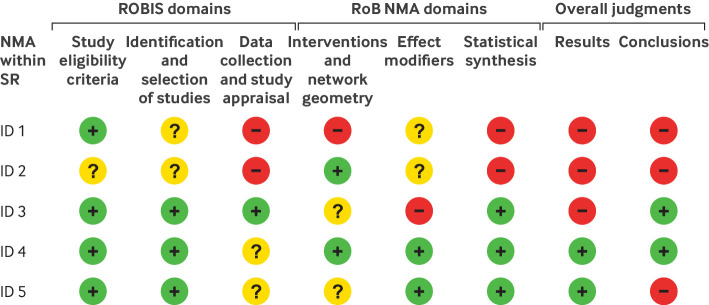
Example format for an overall ROBIS (a tool for assessing risk of bias in systematic reviews) and RoB NMA (Risk of Bias in Network Meta-Analysis) assessment. ID=identification; SR=systematic review

An overall judgment can be done at the level of the results or conclusions of the NMA, or both. Which of these the assessor is interested in will depend on the purpose of the assessment of risk of bias and whether the assessor used the results or the conclusions of the review. For the overall judgment, risk of bias at the systematic review level (eg, with an appropriate tool, such as ROBIS or AMSTAR 2) is combined with the domain level RoB NMA tool judgments to determine whether the findings from the NMA as a whole are at risk of bias. When merging a ROBIS assessment with a RoB NMA assessment, the assessor will have to consider their judgments of the first three domains of the ROBIS tool as well as the three domain level judgments of the RoB NMA tool. Figure 5 has a suggested tabular format for an overall assessment of risk of bias and appendix C has a second suggested format. Phase 3 of the ROBIS tool is omitted when the RoB NMA tool is used with the ROBIS tool. The ROBIS tool is performed at the systematic review level whereas the RoB NMA tool is used for each NMA separately, so in a systematic review with NMA, one ROBIS assessment but multiple RoB NMA assessments will be conducted.

Based on the responses to the signalling statements in each of the domains, an assessor first makes a domain level decision about the risk of bias in the NMA results overall. If all domains are judged at low risk, the NMA result as a whole can be judged at low risk. If high risk of bias was assigned to each of the five domains, the NMA result should generally be judged to be at high risk of bias. Some concerns in multiple domains may make assessors decide on an overall judgment of high risk of bias for that result. The risk of bias in results should be the focus if the NMA results are used in decision models, but the risk of bias in conclusions should be the focus if the NMA conclusions are to be used in decision making.

### Bias in results of NMA

For this section, assessors are asked to consider whether potential bias in the estimated intervention effects (ie, NMA results) was present. Responses for this assessment are low risk of bias, high risk of bias, or some concerns. If all domains were judged at low risk of bias, a judgment of low risk of bias should generally be made. Otherwise, the assessor should decide between some concerns and high risk of bias, depending on the combination of responses to the signalling statements.

### Bias in conclusions of NMA

To assess bias in the conclusions of the systematic review with an NMA, assessors are asked to assign a judgment of concerns or no concerns. Hence the assessor must determine whether in their conclusions, NMA authors dealt with all of the limitations identified in the assessment. Items 3.5, 3.6, and 3.8 should be re-considered when assessing the overall risk of bias in the conclusions because inappropriate modelling choices identified in these items can lead to uncertainty in the results being underestimated or overestimated, which will likely lead to biased conclusions in the NMA. When assessing bias in the conclusions, the importance given to different limitations depends on the context of the NMA being assessed.

The estimated intervention effects could be at high risk of bias and the conclusions of the NMA at low risk of bias. If the estimated intervention effects are at high risk of bias because of the nature of the primary studies, but this risk of bias is carefully taken into account in drawing conclusions, the conclusions of the NMA may be at low risk of bias. If the estimated intervention effects are at high risk of bias because of the conduct of the NMA, however, the conclusions of the NMA are unlikely to be at low risk of bias.

## Discussion

### Summary of the tool and guidance

The RoB NMA tool assesses the possible risk of bias by identifying potential limitations in the way an NMA was conducted, including aspects of how the evidence was assembled that may lead to potential bias in the results of the NMA or the author’s conclusions. The tool has 17 items organised into three domains. We have developed guidance which explains the background, mechanism of bias, and how to assess each item based on each response judgment. Two final statements ask the assessors for a concluding judgment on the risk of bias of the NMA results, and whether they have concerns about the author’s conclusions. The RoB NMA tool is applicable to NMAs of randomised controlled trials, non-randomised studies of interventions, or both. The RoB NMA tool is intended for use with NMAs of healthcare interventions, particularly those reliant on aggregated data (and potentially relevant to individual participant data NMAs), and is relevant to both arm based and contrast based data.

The final phase of the tool combines a systematic review level judgment on the risk of bias with the domain level RoB NMA judgments to determine whether the NMA results and conclusions as a whole are at risk of bias. We recommend that the ROBIS tool is used to assess the systematic review level risk of bias, before a RoB NMA assessment, although we acknowledge that users may prefer other tools, such as AMSTAR 2. Also, the ROBIS tool assesses the risk of bias at the systematic review level, and the RoB NMA tool assesses just one NMA within the review, meaning that several RoB NMA assessments may be necessary to judge the overall risk of bias of the NMAs within the review. These repetitive assessments may burden users because of the workload, time, and effort needed by individuals doing the assessment.

We anticipate that evaluating the potential risks of bias within a systematic review with NMA will be resource intensive in terms of time, skill, cognitive load, and resources. The assessment will most likely be done in collaboration with an NMA expert methodologist or statistician, and an individual with expertise in the clinical, public health, or policy topic under investigation. During pilot training, the median time to complete a RoB NMA assessment was 79 minutes. In a separate study comparing the use of the AMSTAR 2 and ROBIS tools in a cohort of 200 systematic review, the median time to complete the two tools was 59 and 69 minutes, respectively.^91^ Also, it may be necessary to conduct several NMA assessments, because multiple networks may have been conducted within the systematic review. We anticipate that the length of time to complete an assessment will decrease as assessors become more familiar with the tool. Also, assessing subsequent NMAs in a review will require substantially less additional time than the first assessment.

The burden on the assessor can be influenced by various factors, including the complexity of the assessment task, volume of material to be evaluated, level of detail required, and time constraints. A high burden on the assessor could result in challenges such as fatigue, reduced accuracy, and potential delays in completing assessments. If the burden is too high, assessments can be abandoned completely. We aimed to reduce the burden on the assessor by providing clear instructions and combining the RoB NMA assessment with a truncated ROBIS assessment (ie, without the synthesis domain).

### Completeness of reporting and risk of bias judgments

When the comprehensiveness of reporting (ie, reporting quality) in an NMA is poor, assessors may struggle to make a judgment on the overall risk of bias because of increased uncertainty about whether NMA assumptions were met and the evidence appropriately synthesised. Domain level judgments are still required when an NMA presents with missing information for most items. Complete compliance with PRISMA-NMA^14^ is therefore important to make a judgment on low or high risk of bias and avoid unclear judgments (ie, some concerns). An updated PRISMA-NMA will be helpful to users of the RoB NMA tool, and forthcoming work may further increase its value.

### Comparison with domain based tools

The RoB NMA tool has a similar structure to other domain based tools.^23^ ^92^ The tool relies on the evaluation of three individual domains that provide a structured framework to make qualitative decisions on the overall potential sources of bias. As well as domains, and domain level judgments, the tool has 17 items; four items less than the ROBIS tool (n=21) and one more than AMSTAR 2 (n=16). The RoB NMA tool uses signalling statements whereas other tools use signalling questions.^23^ ^92^ The statement format was preferred by the project steering committee because it avoids the use of double negatives often found in signalling questions. We included items related to whether the authors of the study at the synthesis stage appropriately dealt with any identified primary study level bias, potential heterogeneity between studies, reporting biases, and inconsistency identified across comparisons in the network. These concerns are distinct from a GRADE NMA^15^ ^93^ or CINeMA assessment^16^ done by NMA authors that aim to evaluate the credibility in a body of compiled NMA evidence.

In contrast with the AMSTAR 2 tool^10^ and in common with the ROBIS tool,^23^ the RoB NMA tool does not outline specific decision rules for how a user is to judge the domain level judgments or the overall risk of bias in the NMA. We allow subjective rater judgment when assessing domain level and overall bias in the results and conclusions because clinical, public health, and policy interventions will require careful thought and not a standardised or formulaic approach. Also, an overall decision on risk of bias would be subjectively difficult to make when no information is found on most items. A nuanced and customised RoB NMA assessment that considers all items with suspected bias in the tool should be based on the specific clinical, public health, or policy context of interest. Domain based tools, such as the RoB NMA tool, with items that signal to the user what bias may be present, require a careful reading and thoughtful analysis of the NMA to rate the risk of bias adequately, instead of identifying keywords reported in the article, as is usually done in a checklist framework for assessment. A quote from the NMA manuscript or supplement is needed to support the assessor’s judgment of the item for full transparency and to make comparisons with a second assessor more efficient. Items in the RoB NMA tool require that assessors consult the NMA protocols and supplements to make these decisions.

### Future plans

Evaluating the effect of the RoB NMA tool on the risk of bias of future NMAs will be important and could be accomplished by regularly updating the tool to reflect emerging empirical evidence (eg, every five years). Integral to the usability of the RoB NMA tool will be to solicit feedback from end users. Comments from authors on their experience of using the RoB NMA tool are encouraged, for example, by contacting the RoB NMA tool team. The information gathered could help to enhance the uptake of the RoB NMA tool, and will be useful in informing future updates.

The RoB NMA tool has face validity and has been pilot tested, but its performance requires further testing, including a comprehensive assessment of inter-rater reliability across a larger sample of users, especially decision makers. Our future work to test reliability will also include a large number of NMAs assessed by two pairs of assessors who reach consensus by discussion of discrepancies in the items. We will also explore factors that influence agreement between assessors as well as the speed of completion (eg, NMA complexity in terms of number of treatments, outcomes, comparisons, number of included studies, and specific clinical expertise of the reviewer). Judgment is an important part of the assessment of risk of bias and thus perfect agreement is an unrealistic goal. In particular, it may be difficult to reach agreement for NMAs that do not report comprehensive details on methods, data, or results. Variability in agreement across items between users may be high, and detailed instructions for assessors, guiding them on how to reach a rating decision, can help in this regard.

Future software implementations of the tool will also integrate automation of simple step logic completion for specific items. Also, this first version of the tool will benefit from further refinement over time, if gaps or problems are found. Future updates will include extending the tool beyond intervention research to epidemiology, and diagnostic, prognostic, and other types of NMAs.

### Conclusions

An NMA can provide a more complete depiction of the relative and comparative effectiveness of different healthcare interventions, promoting the delivery of optimal and cost effective care. Not all NMAs are conducted according to established and recent guidance, however, and many have a high risk of bias.^94^ ^95^ The RoB NMA tool answers a clearly defined need for a rigorously developed tool for assessing the risk of bias of NMAs for healthcare interventions. The list of items, together with the rationale and example provided for each item, gives a framework for users of NMAs that aims to determine its trustworthiness. The RoB NMA tool enables decision makers to assess and interpret the biases of NMAs, and can help them choose the most trustworthy evidence for patients, public health, and policy formulation. Widespread dissemination and uptake of the RoB NMA tool will hopefully reduce biases in NMAs over time.

By using the RoB NMA tool to identify NMAs with a low risk of bias, decision makers can enhance the quality of guidelines, health technology assessments, funding decisions, and policy or clinical recommendations. Healthcare commissioners and research funders can use NMA results to guide resource allocation by identifying high quality interventions that offer the best balance of effectiveness and cost. Peer reviewers and editors can use the RoB NMA tool to evaluate submitted NMAs to their journals, ensuring that published research meets high standards of credibility and transparency.

## Data Availability

The datasets used or analysed during this study are available freely at https://osf.io/f2b5j/.

## Web extra

Extra material supplied by authors

Appendix A: Methods used to develop the RoB NMA tool

Appendix B: Three RoB NMA tool example assessments

Appendix C: RoB NMA tool template

## Notes

Contributors: CL conceived of the study, recruited participants, analysed the piloting data, and drafted the manuscript. CL, JPTH, IRW, SD, A-AV, BH, JMW, PFW, and ACT provided intellectual input into developing the tool content. CL, IRW, A-AV, ACT, JMW, and BH pilot tested NMAs with the tool. All authors revised, edited, and approved the final manuscript. ACT and CL are the guarantors. The corresponding author attests that all listed authors meet authorship criteria and that no others meeting the criteria have been omitted.

Provenance and peer review: Not commissioned; externally peer reviewed.
