# Correction: Risk of Bias in Network Meta-Analysis (RoB NMA) tool

**DOI:** 10.1136/bmj.r673

**Published:** 2025-04-04

In this paper by Lunny and colleagues (*BMJ* 2025;388:e079839, doi:10.1136/bmj-2024-079839, published 18 March 2025), there was a presentation error in figure 1, which has since been corrected in the article and PDF.

