# Supplementary material for: Risk of Bias in Network Meta-Analysis (RoB NMA) tool
Source: BMJ. 2025 Mar 18;388:e079839. doi: 10.1136/bmj-2024-079839 (PMC11915405; doi:10.1136/bmj-2024-079839)
Supplement: Supplementary file 1 — Appendix A: Methods used to develop the RoB NMA tool [file lunc079839.ww1.pdf]

APPENDIX 1: PILOT TESTING

METHODS
Ethics approval for the pilot testing of the tools was approved by Unity Health Toronto (REB 23-173). Our protocol was registered at https://osf.io/d25wj/ (DOI 10.17605/OSF.IO/D25WJ).

PILOTING RESULTS
Description of Raters
Twenty-one raters gave some form of evaluation of the RoB NMA tool. Of these 18 raters assessed the RoB NMA tool with either an NMA of their choosing (n=12) or assessed the NMA By Crequit et al. (n=7). One rater assessed their own published NMA. Ten of the 21 individuals were NMA experts, five were authors of NMAs, and six had no experience or training with NMAs in general. Half of them worked with their fields for 5 to 10 years, and the other half more than 15 years. All raters had formal training in systematic reviews (i.e., they were either methodologists, had taken a university course or attended a Cochrane workshop in systematic reviews). Four raters were policymakers and worked for: government (n=2), a research hospital (n=1), and a HTA consulting firm (n=2). Two were PhD students in evidenced-based medicine. One rater worked for the pharmaceutical industry. Half of the raters worked for organisations that produced five or more systematic reviews per year, and four produced one to two per year. Eleven assessors had previously used ROBIS or AMSTAR (v1 or 2) to assess systematic reviews. Twelve had used the Cochrane RoB 1.0 or 2.0 tool or ROBINS-I to assess primary studies.

Description of the assessed NMAs
The raters assessed 13 systematic reviews using the RoB NMA tool. The characteristics of the NMAs can be found in **Table 1**. Three were from China and Australia each, and the rest were from singular countries. Ten out of 13 NMAs had published or registered with number (e.g. in PROSPERO) and six used a Bayesian framework. Only one NMA included both RCTs and non-RCTs, and the rest included only RCTs. Included studies ranged from 6 to 522. Just over half of the NMAs examined drug interventions (n=6/13), with the rest examining behavioral/psychological (n=5/13) and surgical (n=2/13) interventions.

Table 1. Characteristics of assessed NMAs (n=13)

First Author Year	Journal	# of authors	Country	Condition	RCTs or Both	Included studies	Intervention type	Bayesian or frequentist	Protocol registered
Samara et al, 2020	Acta Psychiatrica Scandinavica	7	Germany	Nervous system	RCTs	53	Drugs	NA (disconnected network)	Yes
Thakker 2016	Pharmacoepidemiology and drug safety	5	India	Endocrine, nutritional and metabolic diseases	RCTs	29	Drugs	Frequentist	No
de Zoete 2021	British Journal of Sports Medicine	5	Australia	Musculoskeletal system and connective tissue	RCTs	40	Behavioral/psychological	Frequentist	Yes
Cipriani 2018	The Lancet	18	UK	Mental and behavioural disorders	RCTs	522	Behavioral/psychological	Bayesian	Yes
Armstrong 2022	Dermatology and Therapy	13	USA	Skin and subcutaneous tissue	RCTs	86	Drugs	Bayesian	No

Créquit 2017	BMC Medicine	7	France	Neoplasms	RCTs	102	Surgery	Bayesian	Yes
Yang 2023	Annals of Hematology	7	China	Musculoskeletal system and connective tissue	RCTs	6	Surgery	Bayesian	No
Liu 2020	Microbiology Spectrum	19	China	Certain infectious and parasitic diseases	Both	17	Drugs	Frequentist	Yes
Tsikopoulos 2016	British Journal of Sports Medicine	3	Greece	Musculoskeletal system and connective tissue	RCTs	22	Drugs	Bayesian	Yes
Ho 2022	BMJ	9	Australia	Musculoskeletal system and connective tissue	RCTs	97	Behavioral/psychological	Frequentist	Yes
Kishi 2023	Molecular Psychiatry	7	Japan	Mental and behavioural disorders	RCTs	34	Drugs	Bayesian	Yes
Owen 2020	British Journal of Sports Medicine	8	Australia	Musculoskeletal system and connective tissue	RCTs	70	Behavioral/psychological	Frequentist	Yes
Sun 2021	Journal of Back and Musculoskeletal Rehabilitation	5	China	Musculoskeletal system and connective tissue	RCTs	31	Behavioral/psychological	Frequentist	No

Risk of bias assessments

The vast majority of NMAs results had overall risk of bias assessments of high risk (n=7/13) or low risk (n=4/13) with two NMAs assessed as some concerns (n=2/13). For the conclusions, the majority of NMAs were judged as concerning (n=9/13), and the other 4 as no concerns (**Table 2**). **Table 2** provides details on the risk of bias assessments for the individual domains. Details of the NMAs overall and by risk of bias assessments are provided in Appendix A.

Table 2. Risk of bias assessments by domain and overall judgment (n=11)

	**Domains**			**Overall judgment**	
**Domain**	**Interventions and network geometry**	**Effect modifiers**	**Synthesis**	**Results**	**Conclusions**
Samara et al, 2020	Low risk	High risk	High risk	Some concerns	Concerns
Thakker 2016	Low risk	High risk	High risk	High risk	No concerns
de Zoete 2021	Low risk	Low risk	Low risk	Low risk	Concerns
Cipriani 2018	Low risk	Low risk	Some concerns	Low risk	Concerns
Armstrong 2022	Low risk	Low risk	Some concerns	Some concerns	Concerns
Créquit 2017	Low risk	High risk	High risk	High risk	Concerns
Yang 2023	High risk	High risk	High risk	High risk	No concerns

Liu 2020	Low risk	Low risk	Low risk	Low risk	Concerns
Tsikopoulos 2016	High risk	High risk	High risk	High risk	No concerns
Ho 2022	Low risk	Low risk	Low risk	Low risk	Concerns
Kishi 2023	Low risk	Low risk	High risk	High risk	No concerns
Owen 2020	Some risk	High risk	High risk	High risk	Concerns
Sun 2021	High risk	High risk	High risk	High risk	Concerns

* The risk of bias assessments presented here are based on the steering committee's consensus.

The domains that were most frequently rated as low risk of bias was Interventions and Network Geometry (Domain 1).

Interrater agreement

Interrater agreement using a weighted Cohen/Conger's kappa for the NMAs is presented by item, domain and overall judgment in **Table 3**. Items 1.2 and 1.4 had perfect agreement across the 18 raters who assessed 13 NMAs. Items 3.1 and 3.5 did not reach agreement. Agreement for the domains 2 and 3 was considered moderate, as was the overall results and conclusions judgments.

Table 3. Interrater agreement on risk of bias assessments (n=13)

**Domain, item and overall judgment**	**Percentage agreement**	**IRR agreement, weighted κ**	**Interpretation**
**Domain 1 – Interventions and network geometry**			
1.1 All interventions and their comparators included in the NMA are reasonable alternatives for the whole target population	0.86	0.37	Fair
1.2 All eligible *interventions* were included in the network	1.0	1.0	Perfect agreement
1.3 Interventions were appropriately grouped into nodes in the network	0.81	0.08	Slight
1.4 All compared interventions were connected through a suitable chain of within study comparisons	1.0	1.0	Perfect agreement
Domain 1 Judgment	0.94	0.84	Almost perfect agreement
**Domain 2 – Effect modifiers**			
2.1 Outcome definitions and timepoints were similar across direct comparisons in the network	0.73	0.36	Fair
2.2 Effect-modifying participant characteristics were similar across direct comparisons in the network	0.95	0.88	Almost perfect agreement
2.3 Effect-modifying study characteristics were similar across direct comparisons in the network	0.81	0.61	Substantial
2.4 If F/PF to 2.1, 2.2 or 2.3: The analysis appropriately addressed the differences in effect modifiers across the network	0.82	0.60	Moderate
Domain 2 Judgment	0.81	0.60	Moderate
**Domain 3 – Statistical synthesis**			

3.1 No publication bias or other selective non-reporting biases were suspected	0.62	0	Less than chance
3.2 All pre-defined analyses, and only those analyses, were reported, or discrepancies were explained	0.83	0.48	Moderate
3.3 Biases in primary studies were minimal or addressed in the synthesis	0.66	0.33	Fair
3.4 Appropriate methods were used to handle multi-arm studies	0.73	0	Less than chance
3.5 Appropriate assumptions were made about homogeneity or heterogeneity of effects within comparisons	0.69	0	Less than chance
3.6 There was no evidence of conflict between direct and indirect estimates of the same effect	0.81	0.43	Moderate
3.7 If F/PF to 3.6: Conflicting results between direct and indirect evidence were adequately addressed	0.89	0.69	Substantial
3.8 If a Bayesian analysis was performed, the choice of prior distributions was appropriate	0.86	0.61	Substantial
Domain 3 Judgment	0.77	0.41	None to slight
**Overall Judgments**			
Overall risk of bias of the results	0.86	0.65	Substantial
Overall risk of bias of the conclusions†	0.78	0.59	Moderate

Survey results

Seventeen out of 19 assessors filled in our user survey. When asked which items were the hardest to assess, four assessors said items that required clinical judgement or content expertise (e.g. in domain 1) were the most difficult to evaluate. The raters commented:

- 3.3. What does “biases were addressed” mean?
- Item 3.6 about inconsistency as it does not clearly state how to assess it in case there is inability to evaluate inconsistency
- Selective reporting is always hardest, because you have to check registration/protocol against methods, and then against results
- The expression of the level of concern for each domain. I guess it would help if some kind of algorithm was proposed
- Item 2.1, 2.2 and 2.3. These domains are related on how great was the author in reporting information. In particular, item 2.1 “Inspection of the outcome definitions and timepoints used in the included studies should be done at the aggregate level of the direct comparisons”. Very few authors reported these info in detail at aggregate level. As well, for item 2.2. and 2.3, characteristics need to be presented in tabular form at aggregate level (for direct comparison) and not overall. Indeed, it’s difficult hypothesize a judgment when table for comparisons are not present, the judgment would be only an approximation starting from the overall table 1 with the general characteristics.
- Item 3.4 It’s is challenge to judge in the same item two different things: handling multi arm at study level and the adopting of the random model. How can we balance and weight the judgment when there is no study level consideration (eg. split the control group or merge the exp groups) reported but random model considered? For newest NMA there will probably be No Info as the code for WinBugs, and other NMA software incorporates these correlations but for oldest NMA?

- Item 3.9. Difficult to find NMA where sensitivity analyses are used to analyze the robustness of results. If no planned, should we penalize the NMA?
- Bias in the conclusion seems to not be linked to the whole risk of bias NMA assessment. Indeed, in just one item assessors are asked to assign a "Concern" or a "No concern" judgment considering the limitations at the different levels: (i) the primary study and (ii) outcome levels (e.g., bias in primary studies), and (iii) at the systematic review with NMA-level (e.g., incomplete retrieval of identified research, reporting bias). It's not easy to weight a unique judgement. It's not clear if the judgement should just focus on what authors reported in the limitation section.

We also asked the assessors how long it took to complete RoB NMA Tool assessment (excluding reading the NMA. Twelve SRs were timed and the median was 79 minutes and ranged from 30 minutes to 150 minutes. Five of 12 assessors said the time was acceptable (i.e. the workload is balanced by the perceived benefit), four said it was unacceptable, and three said they were undecided.

All assessors said that important items were included. One assessor commented that an item devoted to publication bias is needed (which is addressed by item 3.1 but obviously the item needs to be clarified), and another suggested adding an item about the type of funding in the risk of bias of the NMA. Five respondents said the ease of use of the tool was good, two said it was average, and four said it was poor. Eight raters said the tools had good or very good validity (whether you think the tool helped to distinguish between NMAs of different biases), and two said it was average.

Nine respondents said the clarity of the instruction in the Elaboration document were very good or good, and two said it was average. When asked how they would you suggest improving the tool, five assessors suggested automating the tool and how to determine the judgments for the domains and overall judgments:

- Construct the whole tool using step logic, force raters to make a decision that is binary and unequivocal. This is also possible to evaluate wrt reliability
- Maybe integrate some automated completion for repeated columns like the assessment of each domain?
- I look forward to having some feedback regarding the way to inform summary decision for each domains and overall judgments for the NMA.
- Maybe what is the most missing is how to infer the ROB for each domain and in general from the signaling question

The other suggestions were:

- 2.2 and 2.3 effects modifiers: for true or probably true it should be reported a table or box plots presenting findings by pairwise comparisons otherwise it is not easy to make judgement
- The instruction was somewhat unclear and very lengthy. Simplify, simplify . . .
- There could be specific examples and also a more step-by-step guide on how to use tool
- Include more pragmatic example on what an assessor should look for in the text in order to support the judgement.
- It would be useful to uniform the judgement terms. Now we have judgement of item expressed as true/probably true/ etc… then judgement of domain expressed as low/high risk and finally the judgment of last questions expressed as concern or no concern. I would suggest to use low/high risk for domain and final questions in order to avoid confusion
- Maybe asking the assessor to report its predominant expertise: methodological / clinical? Especially for interpreting its assessment of 'clinical similarity'?
- I wonder if it would be important to distinguish the ROB according to the kind of effect that is being assessed. For example for efficacy outcome the risk is to increase the estimate of the effect. But for

example for a safety outcome, restricting the grouping of intervention potentially decrease the power to identify a safety issue?

Of four respondents, two said they preferred statements over questions, and two said they preferred questions.

When asked if they would use the RoB NMA tool again, seven said yes they would, and one respondent said "Using the RoB NMA tool helped to clarify what the limitations of the network meta-analysis were and gave me much more information than I had gained from initially reading the manuscript." Two were unsure about whether they would use it again but gave no explanation as to why.

When asked about any final comments, several respondents answered:

- I found the Explanations and Elaborations document extremely helpful, particularly the "How should this statement be assessed" guidance for each item, and I used this extensively.
- Develop a unique tool, specific for NMA which integrate ROBIS and ROB-NMA tool with no need to switch to one and another.
- There is probably the need for a detailed guideline for authors on how reported information in a NMA to fulfill the highest standard and for assessor on how and where look for this info.
