# Supplementary material for: Risk of Bias in Network Meta-Analysis (RoB NMA) tool
Source: BMJ. 2025 Mar 18;388:e079839. doi: 10.1136/bmj-2024-079839 (PMC11915405; doi:10.1136/bmj-2024-079839)
Supplement: Supplementary file 2 — Appendix B: Three RoB NMA tool example assessments [file lunc079839.ww2.pdf]

Appendix B: Example RoB NMA assessments

KNOWLEDGE TRANSLATION PROGRAM

RoB NMA Tool

Risk of Bias in Network Meta-Analysis Tool

Developed by: Carole Lunny, Ian R. White, Julian PT Higgins, Sofia Dias, Brian Hutton, James T.Wright, Areti-Angeliki Veroniki, Penny Whiting, Andrea C. Tricco

Version 1 (March 2024)

State the specific NMA you will be assessing: Ho EK, Chen L, Simic M, Ashton-James CE, Comachio J, Wang DX, Hayden JA, Ferreira ML, Ferreira PH. Psychological interventions for chronic, non-specific low back pain: systematic review with network meta-analysis. BMJ. 2022 Mar 30;376.

Network and outcome to be assessed: Pain intensity post-treatment

🗒How to use this tool

The RoB NMA tool identifies potential limitations in the way an NMA was conducted, including aspects of how the evidence was assembled that may lead to bias in the NMA's results or conclusions. The tool contains 17 items organised into three domains: interventions and network geometry (Domain 1), effect modifiers (Domain 2), and statistical synthesis (Domain 3). Within each domain there is a series of signalling statements. The response options to the signalling statements are: True (T), Probably True (PT), Probably False (PF), False (F) and No information (NI) judgments. Risk of bias judgments for each domain are made based on the evaluation of the signalling statements. The possible risk of bias judgments at the domain level and the overall results-level are: Low risk of bias, Some concerns, and High risk of bias.

🗒 Using ROBIS with the RoB NMA tool to make a final risk of bias judgment

The final phase of the tool combines the RoB NMA judgments with a systematic review-level risk of bias/quality judgment (e.g. using an appropriate tool like ROBIS or AMSTAR-2) to determine whether the systematic review with NMA as a whole is at risk of bias. When assessing for potential biases, assessors can use ROBIS as it was designed to identify potential biases at the systematic review-level. Using the domain-based bias judgments in ROBIS's first three domains and the three domains of RoB NMA, the assessor then makes an overall judgment about the potential for bias in the one

DOMAINS AND ITEMS	True	Probably True	False	Probably False	No Information	Not Applicable
**DOMAIN 1 - INTERVENTIONS AND NETWORK GEOMETRY**						
**1.1. All interventions and their comparators included in the NMA are reasonable alternatives for the whole target population**		X				
**Quote:** We included studies of people aged 18 years and older, experiencing chronic, non-specific LBP, with or without the presence of leg pain. We defined chronic, non-specific LBP according to guidelines from the UK National Institute for Health and Care Excellence as pain in the back between						

the bottom of the rib cage and buttocks crease with no known pathoanatomical cause, for more than 12 weeks in duration.17 27

Rationale: It seems reasonable that all interventions and their comparators included in the NMA are reasonable alternatives for the whole target population

**1.2. All eligible interventions were included in the network**		X				

Quote: Studies included in review: 77 Articles included in quantitative synthesis; 76 Unique studies reported within articles*; Articles excluded in quantitative synthesis 21; Unique studies reported within articles†20

Rationale: All eligible interventions included.

**1.3. Interventions were appropriately grouped into nodes in the network**		X				

Quote: Furthermore, although the decision to combine exercise, passive therapy, and physiotherapy into one node was pragmatic (that is, reflecting clinical practice), heterogeneity within the node was a potential limitation. However, we concluded that this potential heterogeneity was unlikely to significantly affect study results because most affected studies involved exercise only (36 (82%) of 44 studies with physiotherapy care as a co-intervention and 19 (58%) of 33 with physiotherapy care as a comparison intervention), or exercise delivered with passive therapy (six (14%) of 44 studies and nine (27%) of 33 studies, respectively).

The final network consisted of 17 treatment nodes (table 1, supplementary B). Examples of intervention or approaches that were classified into the respective treatment nodes have been described in the published protocol paper.25 Psychological interventions were clustered into six nodes: behavioural interventions, cognitive behavioural therapies, mindfulness, counselling, pain education, and combined psychological approaches (that is, the delivery of two or more psychological approaches together, in the absence of a non-psychological co-intervention). Comparison interventions were classified as: physiotherapy care, general practitioner care, advice, no intervention, and usual care. Each psychological intervention node, delivered with physiotherapy care as a co-intervention, formed a separate treatment node. Physiotherapy care was the reference comparison intervention. Physiotherapy care was selected because exercise and passive therapies, which are frequently prescribed or used by physiotherapists, were the most frequently investigated comparison interventions in the included studies and because exercise is the most commonly endorsed treatment approach for managing chronic LBP.8 16 To explore potential heterogeneity within the physiotherapy care node, we identified all studies included in the review that involved physiotherapy care (as a non-psychological co-intervention or a comparison intervention) in at least one of the intervention arms. Then, we delineated between the number of studies in which the physiotherapy care node consisted of exercise alone, passive therapy alone, or exercise delivered with passive therapy.

Table 1 | Final treatment nodes included in network meta-analysis
For studies involving two or more interventions classified as the same treatment node, with at least one other comparison intervention available (eg, a study involving three arms, in which two arms were classified as physiotherapy care, and the third arm was classified as pain education), data from the duplicated treatment nodes were pooled and the study was included in the meta-analyses. However, studies that compared only the same type of psychological intervention, without any other comparison interventions (eg, a study involving two arms, where both arms were classified as cognitive behavioural therapy), were excluded from the meta-analyses.

Rationale: The interventions were appropriately grouped with justification.

**1.4. All compared interventions were connected through a suitable chain of within study comparisons**	X					

Quote: Fig 3 | Network plots of physical function and pain intensity at mid-term and long term follow-up. Adv=advice; BT =behavioural therapy; BT +PC=behavioural therapy delivered with physiotherapy care; CBT =cognitive behavioural therapy; CBT +PC=cognitive behavioural therapy delivered with physiotherapy care; CP=combined psychological approaches; CP+PC=combined psychological approaches delivered with physiotherapy care; Csl=counselling; GP=general practitioner care; Mind=mindfulness; Mind+PC=mindfulness delivered with physiotherapy care; NI=no intervention; PC=physiotherapy care; PE=pain education; PE+PC=pain education delivered with physiotherapy care; UC=usual care

Rationale: The network is connected.

JUDGMENT DOMAIN 1 - Concerns regarding the interventions and network geometry

**Low risk of bias**	X	**Some concerns**		**High risk of bias**	

DOMAIN 2 - EFFECT MODIFIERS

**2.1. Outcome definitions and timepoints were similar across direct comparisons in the network**		X				

Quote: We classified data according to the following intervals: pre-intervention (that is, baseline); post-intervention (that is, at the end of treatment or <2 months postintervention); short term treatment sustainability (from ≥2 to <6 months post-intervention); mid-term treatment sustainability (from ≥6 to <12 months postintervention); and long term treatment sustainability (≥12 months post-intervention). An NMA was conducted at each time point separately.

We assessed transitivity by visual inspection of a table containing categorised study characteristics: mode study setting (inpatient, outpatient,

outpatient online only); intervention duration (weeks); mode of study level mean participant age, dichotomised as younger than 50 years or 50 years and older; mode of study level sex distribution, dichotomised as a population of less than 50% of male individuals or 50% or more of male individuals; and outcome scales reported.

For studies reporting two or more measures for pain intensity at a given time point, we used the following hierarchy for extraction: Numeric Rating Scale, Visual Analogue Scale, rating scale for pain intensity from a composite measure of pain intensity (eg, McGill Pain Questionnaire), and other measurement tools.30 31 For studies reporting two or more measures for pain intensity at a given time point, we extracted data according to the following order: average pain intensity (preferred), worst pain intensity, and alternative measures of pain intensity

Rationale: They grouped timepoints to ensure similarity

**2.2. Effect-modifying participant characteristics were similar across direct comparisons in the network**				X		

Quote: We assessed transitivity by visual inspection of a table containing categorised study characteristics: mode study setting (inpatient, outpatient, outpatient online only); intervention duration (weeks); mode of studylevel mean participant age, dichotomised as younger than 50 years or 50 years and older; mode of study-level sex distribution, dichotomised as a population of less than 50% of male individuals or 50% or more of male individuals; and outcome scales reported.

Rationale: Sex and age seem different which can violate the transitivity assumption.

**2.3. Effect-modifying study characteristics were similar across direct comparisons in the network**		X				

Quote: We assessed transitivity by visual inspection of a table containing categorised study characteristics: mode study setting (inpatient, outpatient, outpatient online only); intervention duration (weeks);

Rationale: Study settings seemed similar; was study duration grouped?

**2.4. If F/PF to 2.1, 2.2 or 2.3: The analysis appropriately addressed the differences in effect modifiers across the network**		X				

Quote: We also acknowledge that inconsistency was detected at various time points of analysis for our primary outcomes. If unresolved, the presence of inconsistency can threaten the validity of the NMA results. However, we performed a thorough examination of potential sources within the network (eg, visually inspecting study and patient characteristics to assess transitivity, exploring potential heterogeneity within the physiotherapy care node, and conducting numerous sensitivity analyses and meta-regressions), and we were able to sufficiently identify and resolve the main sources of inconsistency. Interpretation of study findings were made with consideration of the results of both the primary and sensitivity analyses.
Sensitivity analyses were conducted at short term and mid-term follow-up for physical function, which resolved the presence of inconsistency at these time points. We resolved inconsistency at short term follow-up by removing three studies contributing to intransience related to the measurement tools for assessing physical function, for pairwise comparisons showing inconsistency.67 78-80

Owing to heterogeneity of reporting, we were unable to perform meta-regression based on intervention dosage or frequency.
Nonetheless, meta-regression did not suggest that either mean age or proportion of males were effect modifiers (supplementary Y).
In addition, meta-regression based on mean baseline levels of physical function, pain intensity, or fear avoidance did not suggest these factors were effect modifiers (supplementary Y).

Rationale: They did sensitivity analyses and meta-regressions (except where they could not)

JUDGMENT DOMAIN 2 - Concerns regarding the effect modifiers

Low risk of bias	Some concerns	High risk of bias
x		

DOMAIN 3 - STATISTICAL SYNTHESIS

**3.0. The analysis respected within-study randomisation**	X					

Quote: We performed traditional pairwise meta-analyses for all direct comparisons with at least two studies available, and random effects NMA with a frequentist approach to simultaneously combine direct and indirect evidence.

Rationale: The analysis seems appropriate.

**3.1. All eligible results were included in the analysis**	X					

Quote: Dealing with missing outcome data and missing statistics For continuous outcomes, we imputed missing data by converting standard errors, P values, or confidence intervals into standard deviations

Small-study effects were evaluated by visual inspection of comparison-adjusted funnel plots, including only comparisons with at least one study available, for asymmetry.48 We performed meta-regression using the total sample size to detect small-study effects.50

Rationale: Had good plan to deal with missing data, and examined missing studies

Item						
**3.2. All pre-defined analyses, and only those analyses, were reported, or departures were explained**	X					

Quote: Systematic review registration PROSPERO CRD42019138074.
Examples of interventions or approaches that were classified into the respective treatment nodes have been described in the published protocol paper.25 We attempted but were unable to perform a meta-analysis for health related quality of life and safety, owing to heterogeneity of assessment or reporting or both.

Rationale: Protocol registered and pre-determined analyses reported.

Item						
**3.3. Biases in primary studies were minimal or addressed in the synthesis**	X					

Quote: Sensitivity analyses excluding studies with high risk of bias did not substantially affect the results of the global or local inconsistency tests, suggesting studies with high risk of bias were not an important source of inconsistency.
Sensitivity analysis excluding two studies with high risk of bias produced similar effect estimates (1.09, 0.62 to 1.57 for cognitive behavioural therapy with physiotherapy care; and 0.63, 0.07 to 1.20 for pain education with physiotherapy care; supplementary N).

Rationale: Sensitivity analysis produced similar effect estimates.

Item						
**3.4. Appropriate methods were used to handle multi-arm studies**		X				

Quote: For studies involving two or more interventions classified as the same treatment node, with at least one other comparison intervention available (eg, a study involving three arms, in which two arms were classified as physiotherapy care, and the third arm was classified as pain education), data from the duplicated treatment nodes were pooled and the study was included in the meta-analyses. However, studies that compared only the same type of psychological intervention, without any other comparison interventions (eg, a study involving two arms, where both arms were classified as cognitive behavioural therapy), were excluded from the meta-analyses and the network package and network graphs package for the NMA.47 Stata Statistical Software. Release 14. StataCorp LP, 2015.

We performed traditional pairwise meta-analyses for all direct comparisons with at least two studies available, and random effects NMA with a frequentist approach to simultaneously combine direct and indirect evidence.

Rationale: Not sure what packages they used; they collapsed arms together.

Item						
**3.5. Appropriate assumptions were made about homogeneity or heterogeneity of effects within comparisons**		X				

Quote: For both traditional pairwise meta-analyses and NMA, we estimated random effects using the restricted maximum likelihood method, and derived 95% confidence intervals using the Hartung-Knapp-Sidik-Jonkman approach.41
We assumed that the heterogeneity variance across different comparisons within the NMA model were the same.

Rationale:

Item						
**3.6. There was no evidence of conflict between direct and indirect estimates of the same effect**			X			

Quote: However, we detected global inconsistency at short term and mid-term follow-up (supplementary O). At these time points, local inconsistency was detected in four (22%) of 18 pairwise comparisons at short term follow-up, and five (24%) of 21 pairwise comparisons at mid-term follow-up (supplementary P). Global inconsistency of the entire network was assessed by the design-by-treatment interaction model.48 Local inconsistencies were assessed by the Bucher method.49 If global inconsistency was detected, we explored possible causes of inconsistency through sensitivity analyses.

Rationale: The Bucher method is not appropriate to assess inconsistency.

Item						
**3.7. If N/PN to statement 3.6: Conflicting results between direct and indirect evidence were adequately addressed**				X		

Quote: We also acknowledge that inconsistency was detected at various time points of analysis for our primary outcomes. If unresolved, the presence of inconsistency can threaten the validity of the NMA results. However, we performed a thorough examination of potential sources within the network (eg, visually inspecting study and patient characteristics to assess transitivity,
exploring potential heterogeneity within the physiotherapy care node, and conducting numerous sensitivity analyses and meta-regressions), and we were able to sufficiently identify and resolve the main sources of inconsistency. Interpretation of study findings were made with consideration of the results of both the primary and sensitivity analyses.
We resolved inconsistency at short term follow-up by removing three studies contributing to intransience related to the measurement tools for assessing physical function, for pairwise comparisons showing inconsistency.67 78-80 Inconsistency at midterm follow-up was resolved by removal of the same three studies contributing to

Rationale: They conclude no incoherence based on global test. We disagree based on transitivity assumption violated and local inconsistency test. They didn't explore incoherence for pain intensity post-treatment because they erroneously concluded it wasn't present.

**3.8. If a Bayesian analysis was performed, the choice of prior distributions was appropriate**						X

Quote: We performed traditional pairwise metaanalyses for all direct comparisons with at least two studies available, and random effects NMA with a frequentist approach to simultaneously combine direct and indirect evidence.

Rationale: Frequentist method used so not applicable.

**3.9. If appropriate, Sensitivity analyses demonstrated that findings were robust to the statistical model and estimation methods**					X	

Quote: We attempted to perform a sensitivity analysis by excluding studies with a sample size of less than 100; however, this process resulted in the exclusion of 53 (55%) of 97 studies from our systematic review, leading to large changes in our network structure. Therefore, this additional analysis was not performed. Owing to heterogeneity of reporting, we were unable to perform meta-regression based on intervention dosage or frequency.

Rationale: They attempted but could not perform sensitivity analyses.

JUDGMENT DOMAIN 3 – Concerns regarding the statistical synthesis

Low risk of bias	Some concerns	High risk of bias
x		

	Low risk	High risk	Some concerns
**RESULTS**<br>**A. Bias in the results of the systematic review with NMA**			X

Quote: We did not detect global inconsistency at post-intervention,… However, we detected global inconsistency at short term and mid-term followup (supplementary O). At these time points, local inconsistency was detected in four (22%) of 18 pairwise comparisons at short term follow-up, and five (24%) of 21 pairwise comparisons at mid-term follow-up (supplementary P). Global inconsistency of the entire network was assessed by the design-by-treatment interaction model.48 Local inconsistencies were assessed by the Bucher method.49 If global inconsistency was detected, we explored possible causes of inconsistency through sensitivity analyses.

Rationale: They conclude no incoherence based on global test. We disagree based on transitivity assumption violated and local inconsistency test. They didn't explore incoherence for pain intensity post-treatment because they erroneously concluded it wasn't present.

	Low risk	High risk	Some concerns
**CONCLUSIONS**<br>**B. Bias in the conclusions of the systematic review with NMA**	X		

Quote: This systematic review also had some limitations. Although we separated different types of psychological interventions into five broad but distinct categories to minimise heterogeneity, we made a pragmatic decision to combine interventions involving two or more types of psychological approaches into one treatment node. This decision could have resulted in heterogeneity of combinations of psychological interventions included within this treatment node. However, this pragmatic decision allowed us to gain statistical power and provide a simpler framework from which our findings could be translated more easily into clinical practice. Furthermore, our search strategy aimed to include the most common psychological interventions for patients with chronic, non-specific LBP. However, we identified one type of psychological intervention (hypnosis) that matched our inclusion criteria but did not match our predefined decision set for treatment nodes.104 Consensus within the review team resulted in the inclusion of the study in our review, but exclusion from the NMA because of an inadequate number of studies available for pooling. We also acknowledge that inconsistency was detected at various time points of analysis for our primary outcomes. If unresolved, the presence of inconsistency can threaten the validity of the NMA results. However, we performed a thorough examination of potential sources within the network (eg, visually inspecting study and patient characteristics to assess transitivity, exploring potential heterogeneity within the physiotherapy care node, and conducting numerous sensitivity analyses and meta-regressions), and we were able to sufficiently identify and resolve the main sources of inconsistency. Interpretation of study findings were made with consideration of the results of both the primary and sensitivity analyses. The poor and inconsistent reporting of patient involvement in the design or development of the interventions described in the included studies limited our ability to ascertain whether the psychological interventions are considered acceptable to patients in clinical practice. In parallel, the absence of patient advocates involved in the planning and interpretation of the analyses could be considered a limitation of this review. The inherent inability to blind participants in clinical trials involving psychological interventions should also be considered as a potential source of bias (eg, study results could favour psychological interventions, delivered with or without physiotherapy care, over comparison interventions such as usual care, no interventions, or even physiotherapy care alone). In addition, poor and inconsistent reporting of data for socioeconomic factors and comorbidities precluded examination of these factors as potential

Rationale: They acknowledged their limitations, so low risk in the conclusions.

Appendix B: Example RoB NMA assessments

KNOWLEDGE TRANSLATION PROGRAM

RoB NMA Tool

Risk of Bias in Network Meta-Analysis Tool

Developed by: Carole Lunny, Ian R. White, Julian PT Higgins, Sofia Dias, Brian Hutton, James T.Wright, Areti-Angeliki Veroniki, Penny Whiting, Andrea C. Tricco

Version 1 (March 2024)

State the specific NMA you will be assessing: Samara MT, Huhn M, Chiocchia V, Schneider-Thoma J, Wiegand M, Salanti G, Leucht S. Efficacy, acceptability, and tolerability of all available treatments for insomnia in the elderly: a systematic review and network meta-analysis. Acta Psychiatr Scand. 2020 Jul;142(1):6-17.

Network and outcome to be assessed: Total sleep time

How to use this tool

The RoB NMA tool identifies potential limitations in the way an NMA was conducted, including aspects of how the evidence was assembled that may lead to bias in the NMA's results or conclusions. The tool contains 17 items organised into three domains: interventions and network geometry (Domain 1), effect modifiers (Domain 2), and statistical synthesis (Domain 3). Within each domain there is a series of signalling statements. The response options to the signalling statements are: True (T), Probably True (PT), Probably False (PF), False (F) and No information (NI) judgments. Risk of bias judgments for each domain are made based on the evaluation of the signalling statements. The possible risk of bias judgments at the domain level and the overall results-level are: Low risk of bias, Some concerns, and High risk of bias.

Using ROBIS with the RoB NMA tool to make a final risk of bias judgment

The final phase of the tool combines the RoB NMA judgments with a systematic review-level risk of bias/quality judgment (e.g. using an appropriate tool like ROBIS or AMSTAR-2) to determine whether the systematic review with NMA as a whole is at risk of bias. When assessing for potential biases, assessors can use ROBIS as it was designed to identify potential biases at the systematic review-level. Using the domain-based bias judgments in ROBIS's first three domains and the three domains of RoB NMA, the assessor then makes an overall judgment about the potential for bias in the one

DOMAINS AND ITEMS	True	Probably True	False	Probably False	No Information	Not Applicable
**DOMAIN 1 - INTERVENTIONS AND NETWORK GEOMETRY**						
**1.1.** All interventions and their comparators included in the NMA are reasonable alternatives for the whole target population		X				
**Quote:** "In our meta-analysis, broad inclusion criteria were applied for example insomnia or sleep disorder...as long as all patients did not suffer from the same comorbid medical condition, which is particularly important for reasons of transitivity ...."						

- Melatonin (seven trials), nitrazepam, triazolam, and zolpidem (five trials each),
- Appendix 6: List of included studies lists how each of the participants were diagnosed

Rationale: The diagnoses of patients is different across studies, although it is likely that participants could be randomised to each of the drug interventions as they are over the counter drugs based on age and insomnia symptoms.

**1.2. All eligible interventions were included in the network**		X				

Quote: All available interventions were included. Minimum duration of RCTs was set at 5 days for drug interventions; for non-drug interventions, the study duration criterion did not apply. For each outcome, some of the drugs, although included in the systematic review, were not included in the network meta-analysis because either they were not connected to the network or they had no usable data. For all interventions, pairwise meta-analytic results are presented in Appendix S10. The drug involved in most comparisons was melatonin (seven trials), followed by nitrazepam, triazolam, and zolpidem (five trials each), whereas few trials were available for most other drugs. The remaining 10 RCTs examined the efficacy of other interventions such as acupressure, auricular acupuncture, magneto-auriculotherapy (MAT), laser auriculotherapy (LAT), brief behavioral therapy, hand bath plus massage, massage, mindfulness-based stress reduction program, tart cherry juice, and therapeutic touch. These non-drug interventions were disconnected from the network for all outcomes; therefore, only the results of their pairwise meta-analyses are presented. Moreover, many other drug interventions, such as bromazepam, mirtazapine, and quetiapine which are routinely used to treat insomnia in the elderly, had no available RCT.

Rationale: Even though they could not include all intervention in the network, they did use them in pairwise MA. More transparency about which were excluded would be better.

**1.3. Interventions were appropriately grouped into nodes in the network**		X				

Quote: For drug trials, the network plots of eligible comparisons for the primary outcomes are presented in Figs 2 and 3a and b (see below);

Rationale:
1) intervention kept as separate nodes, so no grouping;
2) Some concerns because doses not grouped based on high vs low --especially for harms. Also, food suppl contains melatonin, and melatonin was a separate node. Poorly reported.

**1.4. All compared interventions were connected through a suitable chain of within study comparisons**	X					

Quote: Fig. 2. Network plot for the outcome ‘total sleep time’. The size of the nodes corresponds to the number of participants assigned to each treatment. Treatments with direct comparisons are linked with a line; its thickness corresponds to the number of trials evaluating the comparison. For each outcome, some of the drugs, although included in the systematic review, were not included in the network meta-analysis because either they were not connected to the network or they had no usable data.

Rationale: Yes, interventions as separate nodes. Network plot in Fig 2**.**

JUDGMENT DOMAIN 1 - Concerns regarding the interventions and network geometry

Low risk of bias	Some concerns	High risk of bias
x		

DOMAIN 2 - EFFECT MODIFIERS

**2.1. Outcome definitions and timepoints were similar across direct comparisons in the network**				X		

Quote: "The few studies available per comparison did not allow firm conclusions about the absence of imbalance in effect modification and in most comparisons only one study was available (Appendix S4). Consequently, the plausibility of the transitivity assumption could not be evaluated."
Appendix 4: Assessment of transitivity "we assessed whether the trials included in the NMA were on average similar in terms of characteristics that might modify the treatment effect (so that the transitivity assumption is plausible)."
"Figure 4.2.1: Boxplot for distribution of study duration across interventions for all studies"

Rationale: No consideration of doses and their timepoints for when the drug start to work. Some of the drugs may have different time that it starts working. Baseline sleep time varied from a min of 231 min to 438 max and median of 313 min.

**2.2. Effect-modifying participant characteristics were similar across direct comparisons in the network**			X			

Quote: "-In our analysis we deemed the following parameters as possible confounders: age, study duration, total sleep time at baseline and sleep quality at baseline."
“---The few studies available per comparison did not allow firm conclusions about the absence of imbalance in effect modification and in most comparisons only one study was available (Appendix S4). Consequently, the plausibility of the transitivity assumption could not be

evaluated."
"Figure 4.2.2.: Boxplot for distribution of age across interventions for studies with data for the primary outcome total sleep time"
-"Figure 4.3.2: Boxplot for distribution of total sleep time at baseline across interventions for studies with data for the primary outcome total sleep time"
“--In our analysis we deemed the following parameters as possible confounders: age, study duration, total sleep time at baseline and sleep quality at baseline. Nevertheless, we could not assess the distribution of the potential modifier sleep quality since different scales were used by the individual studies.”

Rationale:
1) assessing age and severity only
2) Severity has problems ----Baseline sleep time for food supplement seems very high (432 min) when an 8 hour sleep = 480 min?
--range for baseline sleep time is min 234 to max 432 min
--range of study duration is 3 to 168??
--The boxplots shows the distribution across all interventions, not direct comparisons

**2.3. Effect-modifying study characteristics were similar across direct comparisons in the network**				X		

Quote:
"-In our analysis we deemed the following parameters as possible confounders: age, study duration, total sleep time at baseline and sleep quality at baseline."
--"Removing crossover studies or ... did not substantially change the results"
--manuscript "we found indication for high risk of bias for selective reporting in 23 studies (44.9%)."
"The assessment of confidence in the estimates using CINeMA was very low, primarily due to within-study bias and across-studies bias"...
Figure 4.2.1: Boxplot for distribution of study duration across interventions for all studies

Rationale:
1) study duration is a confounder. Duration of the studies varied widely from a min of 5 days to a max of 90 days with a median of 21.
2) biases in the studies seems to be high.

**2.4. If F/PF to 2.1, 2.2 or 2.3: The analysis appropriately addressed the differences in effect modifiers across the network**				X		

Quote:
"The few studies available per comparison did not allow firm conclusions about the absence of imbalance in effect modification and in most comparisons only one study was available (Appendix S4). Consequently, the plausibility of the transitivity assumption could not be evaluated."
“Removing crossover studies or adding studies with psychogeriatric patients did not substantially change the results (Appendices S8.1 and S8.2 respectively).”
“Subgroup, meta-regression, and some of the sensitivity analyses were not undertaken due to insufficient data.”

Rationale: No analyses to account for biases. They could not do many sensitivity analyses that they had polanned, but did do it for some

JUDGMENT DOMAIN 2 - Concerns regarding the effect modifiers

**Low risk of bias**		**Some concerns**		**High risk of bias**	x

DOMAIN 3 - STATISTICAL SYNTHESIS

**3.0. The analysis respected within-study randomisation**	X					

Quote: NMAs in a frequentist setting using the R packages meta (31) and netmeta (32).

Rationale: NMAs in a frequentist setting using the R packages meta

**3.1. All eligible results were included in the analysis**				X		

Quote: "These non-drug interventions were disconnected from the network for all outcomes; therefore, only the results of their pairwise meta-analyses are presented."
--appendix 8: "One study, comparing chlormethiazole versus temazepam, was also excluded from this sensitivity analysis because it was disconnected to the rest of the network."
"we found indication for high risk of bias for selective reporting in 23 studies (44.9%)"
"In our NMA, reporting bias was present in a considerable number of studies highlighting that one of the intrinsic difficulties of the insomnia literature is the multitude of possible outcomes, including subjective and polysomnographic ones."

Rationale: Reporting bias was present in a over half the studies.

**3.2. All pre-defined analyses, and only those analyses, were reported, or departures were explained**	X					
**Quote:** 2.2 Differences between protocol and review<br>28. Strategy for data synthesis: For dichotomous outcomes we used odds ratios (ORs) instead of risk ratios (RR).<br>29. Analysis of subgroups or subsets: A sensitivity analysis was added, namely the exclusion of crossover trials.<br>"Subgroup, meta-regression, and some of the sensitivity analyses were not undertaken due to insufficient data."<br><br>**Rationale:** Departures were explained and Rationale provided for not conducting analyses						
**3.3. Biases in primary studies were minimal or addressed in the synthesis**				X		
**Quote:** The assessment of confidence in the estimates using CINeMA was very low, primarily due<br>to within-study bias and across-studies bias, imprecision, and the inability to evaluate the synthesis assumptions (incoherence) (Appendix S12).<br>provide details about randomization procedures and allocation concealment; three studies were single-<br>blind, three were open-label, one used The assessment of risk for bias is presented in Appendix S7. The trial reports often did not a singleblind design for two arms and open-label design for the third arm, and the remaining studies were double-blind. The mean drop-out rate was 8.4% for the studies included in our systematic review, and we found indication for high risk of bias for selective reporting in 23 studies (44.9%).<br><br>**Rationale:** high risk of bias indicated for selective reporting but no other analyses presented to deal with it						
**3.4. Appropriate methods were used to handle multi-arm studies**		X				
**Quote:** Three studies were single-blind, three were open-label, one used a singleblind design for two arms and open-label design for the third arm, and the remaining studies were double-blind.<br>NMAs in a frequentist setting using the R packages meta (31) and netmeta (32).<br><br>**Rationale:**<br>1) There are indeed multiarm studies in this network<br>2) Netmeta can account correctly for multiarm studies, but because of no reporting on this item, we are unsure whether they correctly entered the data when using Netmeta, or if they ignored the multiarm studies altogether<br><br>Netmeta package handles multi-arm studies.<br>No info on how they handled multiarm studies<br>"NMAs in a frequentist setting using the R packages meta (31) and netmeta (32)."						
**3.5. Appropriate assumptions were made about homogeneity or heterogeneity of effects within comparisons**	X					
**Quote:** We used the random effects model and assumed common heterogeneity across<br>all comparisons.<br><br>**Rationale:** Used random effects appropriately. Although they state that clinical heterogeneity is probably high						
**3.6. There was no evidence of conflict between direct and indirect estimates of the same effect**				X		
**Quote:** Inconsistency of the networks was not measurable since there were no or just one or two closed loops of evidence in each network. The few studies available per comparison did not allow firm conclusions about the absence of imbalance in effect modification and in most comparisons only one study was available (Appendix S4). Consequently, the plausibility of the transitivity assumption could not be evaluated.<br><br>**Rationale:**<br>1) Are the loop in this network those from the multi-arm studies? If yes, there is no inconsistency as they are part of the same trial<br>2) If they are too few studies within the loops, we can still assess inconsistency<br>3) they might have decided not to assess inconsistency because there were only 2 loops to assess with few studies (underpowered)<br>4) or they did assess it and the results were inconclusive						
**3.7. If N/PN to statement 3.6: Conflicting results between direct and indirect evidence were adequately addressed**						X
**Quote:** Not reported<br><br>**Rationale:** N/A. The authors state that lack of data is a reason for not conducting appropriate modelling						
**3.8. If a Bayesian analysis was performed, the choice of prior distributions was appropriate**						X
**Quote:** We performed pairwise meta-analyses and NMAs in a frequentist setting using the R packages meta (31) and netmeta (32).						

Rationale: Not applicable.

**3.9. If appropriate, Sensitivity analyses demonstrated that findings were robust to the statistical model and estimation methods**				X		

Quote: Meta-regression and sensitivity analyses for the primary outcome. Subgroup, meta-regression, and some of the sensitivity analyses were not undertaken due to insufficient data. Removing crossover studies or adding studies with psychogeriatric patients did not substantially change the results (Appendices S8.1 and S8.2 respectively). A further sensitivity analysis, comparing interventions as groups if possible, showed that, for total sleep time, the most to the least effective interventions were food supplement, benzodiazepines, chlormethiazole, antidepressants, and z-drugs (on average 62, 41, 40, 27, and 24 min longer sleep time than placebo, respectively), whereas, for sleep quality, food supplement, chlormethiazole, melatonin and melatoninergic agonists, benzodiazepines, antihistamines, z-drugs, and antidepressants performed better (SMDs 1.90, 0.93, 0.70, 0.53, 0.41, 0.31, and 0.29, respectively, Appendix S8.3).

Rationale: Results were different for total sleep time in the sensitivity analysis. Did not do a sensitivity analysis for low vs high risk studies possibly because there were too few studies?

JUDGMENT DOMAIN 3 – Concerns regarding the statistical synthesis

**Low risk of bias**		**Some concerns**		**High risk of bias**	x

	**Low risk**	**High risk**	**Some concerns**
**RESULTS**<br>**A. Bias in the results of the systematic review with NMA**			X

Quote: Meta-regression and sensitivity analyses for the primary outcome. Subgroup, meta-regression, and some of the sensitivity analyses were not undertaken due to insufficient data. Removing crossover studies or adding studies with psychogeriatric patients did not substantially change the results (Appendices S8.1 and S8.2 respectively). A further sensitivity analysis, comparing interventions as groups if possible, showed that, for total sleep time, the most to the least effective interventions were food supplement, benzodiazepines, chlormethiazole, antidepressants, and z-drugs (on average 62, 41, 40, 27, and 24 min longer sleep time than placebo, respectively), whereas, for sleep quality, food supplement, chlormethiazole, melatonin and melatoninergic agonists, benzodiazepines, antihistamines, z-drugs, and antidepressants performed better (SMDs 1.90, 0.93, 0.70, 0.53, 0.41, 0.31, and 0.29, respectively, Appendix S8.3).

Rationale: ROBIS tool used (Domain 1 to 3), There were no concerns from ROBIS because all methods and criteria were described and justified. Any potential limitations were discussed and the conclusions appropriately reflected. In RoB NMA, two of the domains were judged as having some concerns, while only Domain 1 and the ROBIS level were at low risk. Therefore it is appropriate to judge the bias in the results with some concerns overall. (Biases: 1. high risk of bias indicated for selective reporting but no other analyses presented to deal with it; 2. Results were different for total sleep time in the sensitivity analysis)

**CONCLUSIONS**<br>**B. Bias in the conclusions of the systematic review with NMA**	X		

Quote: Finally, results from a meta-analysis cannot be better than those of the studies included. In our NMA, attrition and reporting bias were present in a considerable number of studies, and the issue of resistance to specific antipsychotics that might be used subsequently as comparators in the included trials (another form of sampling bias) could not be addressed directly. In addition, NMA is a relatively new method that has been criticized even more than conventional meta-analysis because it includes indirect evidence, which adds another level of complexity and assumptions. Indeed, the trials in the network were not as well linked (Figure 2) as in the previous NMA of patients with nonrefractory schizophrenia.12 Enough studies examined clozapine, olanzapine, risperidone, and haloperidol, but, for drugs such as fluphenazine, sertindole, and ziprasidone, the body of evidence was small and conclusions on them are not robust… In addition, the lack of statistically significant differences calls into question the hierarchies found by the NMA, so we prefer to emphasize the effect sizes between individual drugs (of which few were significant) rather than the rankings as presented in Figures 3 and 4…

Rationale: All potential sources of bias and limitations, such as being unable to address some, were discussed in the interpretation of the findings by the review authors.

Appendix B: Example RoB NMA assessments

Risk of Bias in Network Meta-Analysis Tool

Developed by: Carole Lunny, Ian R. White, Julian PT Higgins, Sofia Dias, Brian Hutton, James T.Wright, Areti-Angeliki Veroniki, Penny Whiting, Andrea C. Tricco

Version 1 (March 2024)

State the specific NMA you will be assessing: Thakker D, Nair S, Pagada A, Jamdade V, Malik A. Statin use and the risk of developing diabetes: a network meta-analysis. Pharmacoepidemiology and Drug Safety. 2016 Oct;25(10):1131-49.

Network and outcome to be assessed: Developing diabetes

🗒 How to use this tool

The RoB NMA tool identifies potential limitations in the way an NMA was conducted, including aspects of how the evidence was assembled that may lead to bias in the NMA's results or conclusions. The tool contains 17 items organised into three domains: interventions and network geometry (Domain 1), effect modifiers (Domain 2), and statistical synthesis (Domain 3). Within each domain there is a series of signalling statements. The response options to the signalling statements are: True (T), Probably True (PT), Probably False (PF), False (F) and No information (NI) judgments. Risk of bias judgments for each domain are made based on the evaluation of the signalling statements. The possible risk of bias judgments at the domain level and the overall results-level are: Low risk of bias, Some concerns, and High risk of bias.

🗒 Using ROBIS with the RoB NMA tool to make a final risk of bias judgment

The final phase of the tool combines the RoB NMA judgments with a systematic review-level risk of bias/quality judgment (e.g. using an appropriate tool like ROBIS or AMSTAR-2) to determine whether the systematic review with NMA as a whole is at risk of bias. When assessing for potential biases, assessors can use ROBIS as it was designed to identify potential biases at the systematic review-level. Using the domain-based bias judgments in ROBIS's first three domains and the three domains of RoB NMA, the assessor then makes an overall judgment about the potential for bias in the one

DOMAINS AND ITEMS	True	Probably True	False	Probably False	No Information	Not Applicable
**DOMAIN 1 - INTERVENTIONS AND NETWORK GEOMETRY**						
**1.1.** All interventions and their comparators included in the NMA are reasonable alternatives for the whole target population				X		
**Quote:** Mean age of the patients ranged from 53 to 75 years in the studies. The body mass index (BMI) and LDL-cholesterol levels ranged from 23 to 31 kg/m2 and 97 to 192mg/dL, respectively among the studies. Majority of the studies reported the use of other cardiovascular						

medications.

Rationale: Lack of eligibility criteria mentioned here (which was potentially not controlled for) and the participants had significant comorbidities, different gender, etc.

**1.2. All eligible interventions were included in the network**		X				

Quote: We included 27 studies in the NMA; two studies 31,38 compared any statin versus without statin and were excluded from the analysis (Figure 1).

Rationale: There was no info about this but they probably were all included.

**1.3. Interventions were appropriately grouped into nodes in the network**		X				

Quote: Figure 4 shows network of the treatments for the development of diabetes. Each line links the treatments directly compared in the trial. The thickness of the edge is proportional to the mean control group risk for the comparisons included in the network; the width of the circle is proportional to the number of studies involving the specific treatment. No visible difference in the thickness of the edges supports the fulfilment of transitivity assumption of the network.

Rationale: No rationale is given for the grouping of some doses (e.g. simvastatin 20 and 40mg), while other doses are treated as separate nodes (e.g. simvastatin 80mg).

**1.4. All compared interventions were connected through a suitable chain of within study comparisons**	X					

Quote: Figure 4. Network plot of available direct comparisons. Thickness of the edge is proportional to the mean control group risk for the comparisons included in the network; the width of the circle (node) is proportional to the number of studies involving the specific treatment; colour of the edge indicate risk of bias in a comparison (yellow = moderate and green = low)

Rationale: The network is connected.

JUDGMENT DOMAIN 1 - Concerns regarding the interventions and network geometry

Low risk of bias [x] **Some concerns** [ ] **High risk of bias** [ ]

DOMAIN 2 - EFFECT MODIFIERS

**2.1. Outcome definitions and timepoints were similar across direct comparisons in the network**				X		

Quote: The duration of the trials ranged from 3months to 6.1 years, with the median duration being 4.8 years. Mean age of the patients ranged from 53 to 75 years in the studies. The body mass index (BMI) and LDL-cholesterol levels ranged from 23 to 31 kg/m2 and 97 to 192mg/dL, respectively among the studies. Majority of the studies reported the use of other cardiovascular medications. Tables 1 and 2 provide details on the characteristics of the included studies.

Rationale: Timepoints were very different across the studies

**2.2. Effect-modifying participant characteristics were similar across direct comparisons in the network**				X		

Quote: The thickness of the edge is proportional to the mean control group risk for the comparisons included in the network; the width of the circle is proportional to the number of studies involving the specific treatment. No visible difference in the thickness of the edges supports the fulfilment of transitivity assumption of the network.

Rationale: It seems like the authors only thought that control group risk could be the only effect modifier, but many others could potentially violate the transitvity assumption (see item 2.1)

**2.3. Effect-modifying study characteristics were similar across direct comparisons in the network**					X	

Quote: Not reported

Rationale: A judgement cannot be made because there's no information in the manuscript

**2.4. If F/PF to 2.1, 2.2 or 2.3: The analysis appropriately addressed the differences in effect modifiers across the network**				X		

Quote: They did pairwise meta-regression but not NMA meta-regression methods

Rationale: Analyses to address differences in potential effect modifiers were not explored in the network meta-analysis.

JUDGMENT DOMAIN 2 - Concerns regarding the effect modifiers

Low risk of bias		Some concerns		High risk of bias	x

DOMAIN 3 - STATISTICAL SYNTHESIS

**3.0. The analysis respected within-study randomisation**			x			

Quote: In order to estimate network inconsistency, we calculated the difference between indirect and direct estimates in each closed loop formed by the network of trials (using the Bucher method) and their relative 95%CI.

Rationale: They used the Buchter method which is not suitable, and then they found clear evidence of inconsistency.

**3.1. All eligible results were included in the analysis**		X				

Quote: Figure S4 shows the 'comparison adjusted' funnel plot for our network. In this plot, the horizontal axis presents the difference between the study-specific effect sizes from the corresponding comparison-specific summary effect. In the absence of small study effects, the comparison-adjusted funnel plot should be symmetric around the zero line. In our analysis, although small study effects were seen for some of the studies, these comparison-specific studies were symmetrically distributed around the line of no difference.

Rationale: Nine studies were excluded for language. It's likely that some of these would have been excluded anyway for better reasons such as outcomes, disease, intervention, so the amount of evidence missing is probably small. The comparison-adjusted funnel plot doesn't raise any concerns. Although they state that small study differences might be present

**3.2. All pre-defined analyses, and only those analyses, were reported, or departures were explained**						X

Quote: Not reported

Rationale: No protocol

**3.3. Biases in primary studies were minimal or addressed in the synthesis**			X			

Quote: Around 50% of the studies were at 'high' or 'unclear' RoB for attrition bias and for method employed for detection of diabetes.

Rationale: The majority of the studies are judged to be at moderate risk of bias, but no sensitivity analyses have been performed to explore the impact of potential biases.

**3.4. Appropriate methods were used to handle multi-arm studies**						X

Quote: We performed the pairwise meta-analysis using RevMan 5.1®.We then performed a NMA for incidence of diabetes mellitus within a Frequentist framework, assuming an equal heterogeneity parameter tau ( ) across all comparisons.

Rationale: I think the one multi-arm study (Chen 2013) has probably been handled by collapsing three atorvastatin doses into one, but this isn't explained in the manuscript.

**3.5. Appropriate assumptions were made about homogeneity or heterogeneity of effects within comparisons**				X		

Quote: We then performed a NMA for incidence of diabetes mellitus within a Frequentist framework, assuming an equal heterogeneity parameter tau across all comparisons.

Rationale: Unexplained heterogeneity is modelled using a random-effects model. For the NMA, they "assuming an equal heterogeneity parameter tau across all comparisons". Heterogeneity assumed equal across comparisons, but there is no clear evidence against this assumption.

**3.6. There was no evidence of conflict between direct and indirect estimates of the same effect**			X			

Quote: In order to estimate network inconsistency, we calculated the difference between indirect and direct estimates in each closed loop formed by the network of trials (using the Bucher method) and their relative 95%CI. We then examined whether there were any material discrepancies; if the 95%CI did overlap with 1 the hypothesis of consistency was not rejected, as described in Salanti et al.22
There was evidence of statistical and clinical inconsistency in the two triangular loops atorvastatin– placebo–simvastatin and atorvastatin–atorvastatin 80 mg-simvastatin with the ratio of odds ratio ROR 4.4; 95%CI 1.08–18.04 and ROR 4.41; 95%CI 1.08–18.09, respectively.

Rationale: They used the Buchter method which is not suitable, and then they found clear evidence of inconsistency.

**3.7. If N/PN to statement 3.6: Conflicting results between direct and indirect evidence were adequately addressed**			X			

Quote: Not reported

Rationale: The conflict found between indirect and direct evidence was not explored further.

**3.8. If a Bayesian analysis was performed, the choice of prior distributions was appropriate**						X

Quote: We adopted a Frequentist analytic approach, which would be expected to yield identical results as compared with the analyses conducted within a Bayesian framework with non-informative priors as used by Naci et al.16 and Navarese et al.17

Rationale: Frequentist method used

**3.9. If appropriate,** Sensitivity analyses demonstrated that findings were robust to the statistical model and estimation methods			X			

Quote: Not reported

Rationale: Sensitivity analyses to evaluate robustness were not performed.

JUDGMENT DOMAIN 3 – Concerns regarding the statistical synthesis

Low risk of bias		Some concerns		High risk of bias	x

	Low risk	High risk	Some concerns
**RESULTS**<br>**A. Bias in the results of the systematic review with NMA**		X	

Quote: In order to estimate network inconsistency, we calculated the difference between indirect and direct estimates in each closed loop formed by the network of trials (using the Bucher method) and their relative 95%CI.

Rationale: The authors used the wrong methods – the Buchter method for the analysis, and they found inconsistency which was not resolved.

	Low risk	High risk	Some concerns
**CONCLUSIONS**<br>**B. Bias in the conclusions of the systematic review with NMA**		X	

Quote: We found evidence of inconsistency in the atorvastatin–placebo–simvastatin atorvastatin–atorvastatin 80 mg-simvastatin triangular loops; therefore, mixed estimates related to this loop should be interpreted with caution. We assessed the risk of diabetes across all trials and all statins for the CVDs. However, we recognize that this may be inappropriate in this case for several reasons. Different statins and existing co-morbidities may be associated with different risk profiles for developing diabetes. In the context of the aforementioned limitations, our study used the best available evidence to show no potential risk of diabetes with the different classes of statins that were compared. However, large, long-term studies are required to assess this risk and the potential causes, with long-term statin use.

CONCLUSION
Based on the results, statins, as a class, increased the risk of diabetes significantly in the pairwise meta-analysis. Overall, there appears to be a small increased risk of incident diabetes, particularly with more intensive statin therapy, although more data would be valuable to increase the robustness of this interpretation, given that the lower confidence intervals of our study analyses are close to, or just crossing one.

Rationale: From one paragraph to the next they have two very different and opposing conclusions! And The limitations of the review and analyses are not adequately discussed, so the conclusions drawn from the results are questionable.
