# Supplementary material for: Risk of Bias in Network Meta-Analysis (RoB NMA) tool
Source: BMJ. 2025 Mar 18;388:e079839. doi: 10.1136/bmj-2024-079839 (PMC11915405; doi:10.1136/bmj-2024-079839)
Supplement: Supplementary file 3 — Appendix C: RoB NMA tool template [file lunc079839.ww3.pdf]

# Risk of Bias in Network Meta-Analysis Tool

**Developed by**: Carole Lunny, Ian R. White, Julian PT Higgins, Sofia Dias, Brian Hutton, James T. Wright, Areti-Angeliki Veroniki, Penny Whiting, Andrea C. Tricco

**Version 1 (March 2024)**

## State the specific NMA you will be assessing:

## How to use this tool

The RoB NMA tool identifies potential limitations in the way an NMA was conducted, including aspects of how the evidence was assembled that may lead to bias in the NMA's results or conclusions. The tool contains 18 items organised into three domains: interventions and network geometry (Domain 1), effect modifiers (Domain 2), and statistical synthesis (Domain 3). Within each domain there is a series of signalling statements. The response options to the signalling statements are: True (T), Probably True (PT), Probably False (PF), False (F) and No information (NI) judgments.

Risk of bias judgments for each domain are made based on the evaluation of the signalling statements. The possible risk of bias judgments at the domain level and the overall results-level are: Low risk of bias, Some concerns, and High risk of bias.

## Using ROBIS with the RoB NMA tool to make a final risk of bias judgment

The final phase of the tool combines the RoB NMA judgments with a systematic review-level risk of bias/quality judgment (e.g. using an appropriate tool like ROBIS or AMSTAR-2) to determine whether the systematic review with NMA as a whole is at risk of bias. When assessing for potential biases, assessors can use ROBIS as it was designed to identify potential biases at the systematic review-level. Using the domain-based bias judgments in ROBIS's first three domains and the three domains of RoB NMA, the assessor then makes an overall judgment about the potential for bias in the one NMA result (e.g. one outcome from one network).

## DOMAIN 1: INTERVENTIONS AND NETWORK GEOMETRY

| **1.1 All interventions and their comparators included in the NMA are reasonable alternatives for the whole target population** | ⊗ |
|---|---|
| Answer **True** if the review authors provide evidence that all studies include similar participants or that individuals contraindicated for one intervention are excluded from all studies. | ○ |
| Answer **Probably True** if it seems likely on clinical grounds that studies include similar participants or that individuals contraindicated for one intervention are excluded from all studies, although the authors did not show this explicitly. | ○ |
| Answer **Probably False** if there are concerns that some participants would not be eligible for some of the interventions. | ○ |
| Answer **False** if it is clear that some participants would not be eligible for some of the interventions. | ○ |
| Answer **No information** when insufficient data are reported to permit a reasonable judgement to be made. | ○ |

| **1.2 All eligible interventions were included in the network** | ⊗ |
|---|---|
| Answer **T** if no interventions were inappropriately excluded. | ○ |
| Answer **PT** if interventions were appropriately excluded with clear justification given. | ○ |
| Answer **PF** if interventions were excluded with no clear justification to determine if this was appropriate or not. | ○ |
| Answer **F** if interventions *were* inappropriately excluded; that is, excluded based on their likely impact on the results of the NMA, in order to ensure that results agree with *a priori* views about the relative effects of the interventions. | ○ |
| Answer **NI** when insufficient data are reported to permit a reasonable judgement to be made. | ○ |
| **1.3 Interventions were appropriately grouped into nodes in the network** | ⊗ |
| Answer **T** if (i) the rationale for grouping interventions was sound, the approach was pre-specified, and there is no suggestion of important differences in the effects of grouped interventions; or (ii) there was no grouping of different interventions. | ○ |
| Answer **PT** if interventions have been combined for which different effects are possible but unlikely. | ○ |
| Answer **PF** if interventions have been combined which are likely to have different effects. | ○ |
| Answer **F** if interventions have been combined which are known to have different effects. | ○ |
| Answer **No Information** when insufficient data are reported to permit a reasonable judgement to be made. | ○ |
| **1.4 All compared interventions were connected through a suitable chain of within study comparisons** | ⊗ |
| Answer **T** if it is clear that the network is connected, or if the network contained some disconnected interventions but the authors only made comparisons between interventions contained in a connected sub-network. | ○ |
| Answer **PT** if the authors connected the network by adding additional interventions for the purpose of creating indirect evidence on the main comparisons of interest. | ○ |
| Answer **PF** if the authors report that steps were taken to connect disconnected interventions using approaches that involved use of observational data, expert opinion, borrowing of data from related conditions or other such methods. | ○ |
| Answer **F** if the network included single-arm studies or if results are reported for disconnected comparisons. | ○ |
| Answer **NI** when insufficient data are reported to permit a reasonable judgement to be made. | ○ |

| **Concerns regarding the domain-level network characteristics and geometry** | | |
|---|---|---|
| ○ **Low risk of bias** | ○ **Some concerns** | ○ **High risk of bias** |

## DOMAIN 2: EFFECT MODIFIERS

| | |
|---|---|
| **2.1 Outcome definitions and timepoints were similar across direct comparisons in the network** | ⊗ |
| Answer **T** if studies making different comparisons used outcome definitions or timepoints that were identical, or similar enough for intervention effects not to be affected by these differences | ○ |
| Answer **PT** if studies making different comparisons used different outcomes or timepoints and the differences are unlikely to be associated with differences in intervention effect. | ○ |
| Answer **PF** if studies making different intervention comparisons used different outcomes or timepoints and these are likely to result in different intervention effects. | ○ |
| Answer **F** if studies making different intervention comparisons used different outcomes or timepoints and these are known to result in different intervention effects. | ○ |
| Answer **NI** when insufficient data are reported to permit a reasonable judgement to be made. | ○ |
| **2.2 Effect-modifying participant characteristics were similar across direct comparisons in the network** | ⊗ |
| Answer **T** if studies making different comparisons were similar in known and suspected effect-modifying participant characteristics. | ○ |
| Answer **PT** if studies making different comparisons were reasonably similar (or are judged to be reasonably similar) in known or suspected effect-modifying participant characteristics. | ○ |
| Answer **PF** if there is suspicion that effect modifying participant characteristics were not reasonably similar across comparisons. | ○ |
| Answer **F** if there is evidence of important variation across comparisons in known or suspected effect modifying participant characteristics. | ○ |
| Answer **No Information** when insufficient data are reported to permit a reasonable judgement to be made. | ○ |
| **2.3 Effect-modifying study characteristics were similar across direct comparisons in the network** | ⊗ |
| Answer **T** if studies making different direct comparisons were similar in known and suspected effect-modifying study characteristics (including consideration of the nature and direction of any biases). | ○ |
| Answer **PT** if studies making different direct comparisons were reasonably similar (or are judged to be reasonably similar) in most known or suspected effect-modifying study characteristics. | ○ |
| Answer **PF** if there is suspicion that effect modifying study characteristics were not reasonably similar across direct comparisons. | ○ |
| Answer **F** if there is evidence of important variation across direct comparisons in known or suspected effect modifying study characteristics. | ○ |
| Answer **NI** when insufficient data are reported to permit a reasonable judgement to be made. | ○ |

| **2.4 If F/PF to 2.1, 2.2 or 2.3: The analysis appropriately addressed the differences in effect modifiers across the network** | ⊗ |
|---|---|
| Answer **T** if the authors have used acceptable methods to conduct the analysis, such as (i) statistical methods (e.g. meta-regression, subgroup analysis or mixed-effects models) were used that were likely to account for these differences, or (ii) sensitivity analysis demonstrated that the differences were unimportant in the review adjusting for the inclusive set of effect modifiers in question and addressed the comparability of findings with those from unadjusted analyses. | ○ |
| Answer **PT** if analyses were partly explored, but there are remaining differences that were not addressed or if findings from analyses were not adequately considered. | ○ |
| Answer **F/PF** if analyses to address differences in potential effect modifiers were not explored. | ○ |
| Answer **NI** when insufficient data are reported to permit a reasonable judgement to be made. | ○ |

| **Summarising Domain 2 : Concerns regarding domain-level effect modifiers** | | |
|---|---|---|
| ○ **Low risk of bias** | ○ **Some concerns** | ○ **High risk of bias** |

# DOMAIN 3: STATISTICAL SYNTHESIS

| **3.1 The analysis respected within-study randomisation** | ⊗ |
|---|---|
| Answer **T** if (i) within-study comparisons were analysed, or (ii) arm-level or patient-level data were analysed with a model including a fixed effect of study. | ○ |
| Answer **PT** if arm-level or patient-level data were analysed with a model including a random effect of study, and average outcomes were similar across direct comparisons. Also answer PT if this was done in a sensitivity analysis which gave results similar to the main results. | ○ |
| Answer **PF** if arm-level or patient-level data were analysed with a model including a random effect of study, and average outcomes were different across direct comparisons. | ○ |
| Answer **F** if arm-level or patient-level data were analysed ignoring study, or if data were combined across studies before being compared between interventions. | ○ |
| Answer **NI** when insufficient data are reported to permit a reasonable judgement to be made. | ○ |
| **3.2 No publication bias or other selective non-reporting biases were suspected** | ⊗ |
| Answer **T** if (i) the rationale for grouping interventions was sound, the approach was pre-specified, and there is no suggestion of important differences in the effects of grouped interventions; or (ii) there was no grouping of different interventions. | ○ |
| Answer **PT** if the amount of evidence potentially excluded from the NMA is so small that its inclusion would have trivial impact on the findings, or if it is likely that any results not included in the NMA were excluded for reasons unrelated to the findings. | ○ |
| Answer **PF** if it is likely that eligible results were missing from the NMA (either due to their suppression by the study authors or their exclusion by the review authors) and either (i) may be systematically different from the results included in the NMA or (ii) the amount of evidence missing from the NMA is sufficiently large that its inclusion could affect estimated intervention effects from the NMA. | ○ |
| Answer **F** if there is evidence that eligible results were missing from the NMA (either due to their suppression by the study authors or their exclusion by the review authors) and these results were likely to be systematically different from the results included in the NMA, with a sufficient amount of evidence missing that it could affect estimated intervention effects from the NMA. | ○ |
| Answer **No Information** when insufficient data are reported to permit a reasonable judgement to be made. | ○ |
| **3.3 All pre-defined analyses, and only those analyses, were reported, or discrepancies were explained** | ⊗ |
| Answer **T** if it is clear that predefined analyses were followed, for example because they follow a detailed, pre-specified protocol. | ○ |
| Answer **PT** if (i) there is an indication that predefined analyses, and only those analyses were followed, for example because a protocol is insufficiently detailed but the methods implemented are sensible and in accordance with what was written in the protocol; or (ii) no protocol is available but the methods section appears rigorous and all analyses mentioned are addressed in the results; or (iii) all departures from planned methods were justified using reasons unrelated to the observed results. | ○ |
| Answer **PF** if there is no indication that pre-defined analyses were followed, for example because there was insufficient detail about the methods that are likely to have been planned and implemented (e.g. no protocol and insufficient clarity in the methods section). | ○ |
| Answer **F** if is clear that predefined analyses were not followed, or that other analyses were used, and an indication (or evidence) that the departures from the predefined analyses were made because of the results. | ○ |
| Answer **NI** when insufficient data are reported to permit a reasonable judgement to be made. | ○ |

| | |
|---|---|
| **3.4 Biases in primary studies were minimal or addressed in the synthesis** | ⊗ |
| Answer **T** if (i) all studies were assessed as being at low risk of bias, or (ii) sensitivity analyses demonstrated that there was no impact of including studies at higher risk of bias on the results. | ○ |
| Answer **PT** if (i) the proportion of information at high risk of bias was too small for it to impact on the results, or (ii) sensitivity or threshold analyses demonstrated that there was minimal impact of including studies at high risk of bias, or (iii) adjustment approaches were used that are likely to have corrected for biases. | ○ |
| Answer **PF** if (i) the proportion of information at high risk of bias was sufficient for it to impact on the results, and sensitivity analyses did not demonstrate that there was minimal impact of including studies at high risk of bias, or (ii) bias adjustment approaches were used and it is unclear whether they are likely to have corrected for biases, or (iii) risk of bias in the included studies was not assessed. | ○ |
| Answer **F** if (i) there are important biases in primary studies and these have not been addressed by the reviewers, or (ii) sensitivity analyses demonstrate that results are strongly influenced by studies at higher risk of bias. | ○ |
| Answer **NI** when insufficient data are reported to permit a reasonable judgement to be made. | ○ |
| **3.5 Appropriate methods were used to handle multi-arm studies** | ⊗ |
| Answer **T** if there are no multi-arm studies as clearly indicated by descriptions of the included studies. Also answer **T** if [IW1] (a) the data from multi-arm studies were included either as arm-level data or as relative effects with an adequate correlation supplied; and (b) the methods used adequately account for the correlation in random effects from multi-arm studies (if a random effects model was used). | ○ |
| Answer **PT** if NMA software, code, or methods were not fully reported to address the correlation in random effects in multi-arm studies, but other evidence suggests appropriate software and methods were used. | ○ |
| Answer **F/PF** if (i) multi-arm studies were not appropriately represented in the data set, or (ii) the methods chosen did not adequately account for the correlations in random effects or did not indicate the appropriate handling of these correlations. | ○ |
| Answer **No Information** when insufficient data are reported to permit a reasonable judgement to be made. | ○ |
| **3.6 Appropriate assumptions were made about homogeneity or heterogeneity of effects within comparisons** | ⊗ |
| Answer **T/PT** if heterogeneity is not modelled (e.g., using a fixed-effect model for rare events) and this is well justified, or if unexplained heterogeneity is modelled (i.e., random effects model) and there is no evidence in the data against the modelling assumptions. One justification for a particular modelling approach is if a sensitivity analysis is performed using a more appropriate or more general modelling approach and this sensitivity analysis shows results similar to the main results. | ○ |
| Answer **F/PF** if heterogeneity is not modelled and this is not well justified, or if heterogeneity is modelled and there is evidence in the data against the modelling assumptions. | ○ |
| Answer **NI** when insufficient data are reported to permit a reasonable judgement to be made. | ○ |
| **3.7 There was no evidence of conflict between direct and indirect estimates of the same effect** | ⊗ |
| Answer **T** if there were no potential sources of inconsistency in the network (i.e. no closed loops, or loops formed only by the comparisons in a multi-arm study), or if a suitable exploration of inconsistency was applied and there was no indication that inconsistency was present (this would usually require a sizeable evidence base to overcome issues of low power in tests for inconsistency). | ○ |
| Answer **PT** if a suitable exploration of inconsistency was applied and there was no evidence of inconsistency. This may include cases where some estimates are very extreme due to e.g. zero cells, but the overall direction of effect is consistent. | ○ |
| Answer **PF** if there was some evidence of inconsistency but it did not look serious, or there are important concerns that the tests performed had low power to detect inconsistency. | ○ |
| Answer **F** if no information about inconsistency is provided, or there is clear evidence of inconsistency. | ○ |
| Answer **NI** when insufficient data are reported to permit a reasonable judgement to be made. | ○ |

| **3.8 If F/PF to 3.6: Conflicting results between direct and indirect evidence were adequately addressed** | ⊗ |
|---|---|
| Answer **T/PT** if the alternative analyses had a clear justification, and resolved the original conflict. | ○ |
| Answer **F/PF** if the direct MA results were compared with the NMA results or previous literature (e.g., systematic reviews) to address conflicting results, or if the primary studies were randomly excluded until consistency was reached. | ○ |
| Answer **No Information** when insufficient data are reported to permit a reasonable judgement to be made. | ○ |
| **3.9 If a Bayesian analysis was performed, the choice of prior distributions was appropriate** | ⊗ |
| Answer **Not Applicable** if a Bayesian analysis was not conducted (i.e., frequentist analysis). | ○ |
| Answer **T** if (i) prior distributions were used that were clearly non-informative across the range of possible parameter values, or (ii) if informative prior distributions were used and had a clear justification, or (iii) if sensitivity analysis with alternative appropriate prior distributions was performed and showed results similar to the main results | ○ |
| Answer **PT** if off-the-shelf non-informative prior distributions were used. | ○ |
| Answer **F/PF** if informative prior distributions were used that were not clearly justified. | ○ |
| Answer **NI** when insufficient data are reported to permit a reasonable judgement to be made. | ○ |

**Summarizing Domain 3: Concerns regarding the NMA synthesis**

○ **Low risk of bias** ○ **Some concerns** ○ **High risk of bias**

## OVERALL RISK OF BIAS IN THE NMA

| **A. Bias in the results of the NMA** | ⊗ |
|---|---|
| Answer **High risk** of bias if any domain from either the ROBIS assessment or the ROB NMA assessment is judged at high risk of bias**.** | ○ |
| Answer **Low risk of bias** if all domains from either the ROBIS assessment or the ROB NMA assessment is judged at low risk of bias. | ○ |
| Answer **Some concerns** if any individual domain from either tools are judged as “Some concerns”, or “High risk of bias”. | ○ |

**B. Bias in the conclusions of the NMA**

Assessors can also make an overall judgment of the bias in the authors’ conclusions by assessing the interpretation of the findings. This examines whether the concerns identified were addressed; whether the relevance of studies was considered (ROBIS Phase 1); and whether the authors avoided emphasizing results based on statistical significance. Items 3.5, 3.6 and 3.9 should be re-considered when assessing the overall risk of bias in the conclusions, because inappropriate modelling choices identified in these items can lead to uncertainty in the results being underestimated, which is likely to lead to bias in the conclusions of the NMA.

○ **Concerns** ○ **No concerns**
